# First online field measurements of chlorothalonil volatilisation using proton transfer mass spectrometry and inverse modelling

**DOI:** 10.1038/s41598-025-13898-0

**Published:** 2025-08-19

**Authors:** Benjamin Loubet, Carole Bedos, Julien Kammer, Céline Decuq, Florence Lafouge, Baptiste Esnault, Raluca Ciuraru, Sandy Bsaibes, Pauline Buysse, Enrique Barriuso, Maria Raffaella Vuolo, Valérie Gros

**Affiliations:** 1 ECOSYS, Université Paris-Saclay, INRAE, AgroParisTech, Palaiseau, France; 2 Now at LCE, Université Aix Marseille, CNRS, Marseille, France; 3 LSCE, CEA, CNRS, Université Paris-Saclay, Gif-sur-Yvette, France; 4 Food and Agriculture Organization of the United Nations, Rome, Italy

**Keywords:** Environmental chemistry, Environmental impact

## Abstract

Pesticide usage has been expanding since the 1950s. Their use has affected human and environmental health negatively for decades. Recent studies have shown that gaseous pesticide diffusion in the atmosphere, known as volatilisation, may contribute to the spreading of pesticides in the environment. Although pesticide volatilisation is a known process, it has been scarcely measured, especially for periods beyond a few days after pesticide application. The reason is that pesticide concentration measurement, done mainly by offline gas chromatography-mass spectrometry, is challenging to deploy in the field for long-term studies. In this study, we report the first online concentration measurements of the fungicide chlorothalonil over a wheat field for several weeks after application, using a highly sensitive proton transfer reaction, quadrupole injection, time of flight, mass spectrometer (PTR-QI-TOF-MS). The main finding of this study is that chlorothalonil volatilisation, computed by inverse dispersion modelling, was sustained for more than three weeks after application. Overall, we evaluate that from 20 to 50% of the applied quantity of this fungicide may be lost by volatilisation to the atmosphere, which represents a quantity much higher than the currently accepted consensus. The analysis of the high-time resolution volatilisation dynamics further shows that volatilisation is the primary process of chlorothalonil dissipation over the 3 weeks, while penetration and photodegradation would be the dominant dissipation processes during the first 5 days. Our findings indicate that chlorothalonil gaseous volatilisation may be the most significant contributor to atmospheric exposure to this pesticide and its transfer to the environment. If we extrapolate our results to other volatile pesticides, we expect pesticide volatilisation to be a much larger exposure route than expected for humans and the environment.

**Supplementary Information:**

The online version contains supplementary material available at 10.1038/s41598-025-13898-0.

## Introduction

Pesticide usage has increased worldwide since the 1950s, levelling to approximately 4 Mt since 2010. In 2018, Asia was the most common consumer (52%), followed by the Americas (32%) and Europe (12%), while Africa and Oceania consumed only 2% of each of the global products^1^. At that time, pesticide usage was divided worldwide into 30% herbicides, 13% fungicides, 10% insecticides, and 45% other kinds^1^. One fungicide used worldwide is chlorothalonil (1,3-benzenedicarbonitrile, 2,4,5,6-tetrachloro), a broad-spectrum non-systemic organochloride fungicide derived from benzene. It is an organic compound with the chemical formula C_8_Cl_4_N_2_ and a molar mass of 265.911 g mol^−1^. In 2018, the year of this study, 1.7 kT was produced in France, with constant production growth of 0.2 kT per year since 2012 (Supp. Table1)^2^. In 2014, chlorothalonil was used at least once per crop cycle for 43% of French national wheat production, which amounted to 2 Mha. In the USA, chlorothalonil was consistently measured in 16% of crop residues from 1994 to 2020^3^. Its use was banned in 2019 in the EU (Regulation EC No 1107/2009)^4,5^ but it is still allowed in most countries worldwide and is even exported by Europe^6^.

After spraying onto the field, chlorothalonil can leach into the soil, be degraded, absorbed by plants and animals^7^or volatilised^8^. It is degraded by soil bacteria^9^ but also through hydrolysis, photolysis, and photodegradation^10,11^. Its currently reported lifetime in the environment is less than 60 days^4^. However, the common chlorothalonil metabolite R471811 has a half-life of several years^4^. Despite the chlorothalonil ban in 2019 in Europe, studies in 2020 and 2023 in Switzerland^12^ and France^13^ reported that R471811 was found in 50% and 100% of the surface and groundwater samples, respectively, and above the quality threshold of 100 ng/L in more than 30% of the samples. This shows that even if chlorothalonil is degraded, its degradation products may persist for a long time in the environment. Residues of chlorothalonil and its metabolites in animal tissues have been observed in laboratory studies at various times after acute oral or dermal exposure^9^. Effects such as kidney and stomach lesions have also been reported on animal tissues^14,15^. Acute exposure toxicity is even more pronounced for the inhalation route, which is responsible for severe adverse effects ranging from nasal staining to lethal respiratory syndrome^4,16^. Chronic exposure through oral administration can lead to different lesions, including ulcers, adenomas, and carcinomas^16^. For those reasons, chlorothalonil was classified as “possibly” or “likely” carcinogenic to humans by the IARC and US-EPA in 1999^17,18^. More recently, occupational exposure has been investigated in epidemiological and retrospective-prospective cohort studies, which supports the hypothesis that chlorothalonil may be causally linked to cancer^19–22^.

As for most pesticides, data on gaseous chlorothalonil volatilisation following application are scarce despite its suspected effects through inhalation or dermal exposure. On the contrary, chlorothalonil atmospheric concentrations were measured in the past: they were first reported in the late 1990s^23^ at concentrations of 1 to 300 ng m^−3^ following application near the applied field. Recent studies have reported ranges between 140 and 800 ng m^−3^ measured over a short time (30 min) in an agricultural context in the USA and Canada^24–26^. Kruse-Plaß et al.^27^. reported maximum, six-month average concentrations of 3 ng m^−3^ in Germany in 2019, while Coscolla et al.^28,29^. reported concentrations averaging 23 ng m^−3^ over a year in a rural area in central France, with a maximum above 1000 ng m^−3^ over a week during the application period. Habran et al. (2024)^30^ reported concentrations ranging from 0.1 to 12.36 ng m^−3^ in Wallonia, Belgium, in May 2016. These studies showed that chlorothalonil was widely present in the environment, even in remote natural regions far from treated areas, as in the Bavarian forest^31^. Similarly, in France, the chlorothalonil concentration has been reported at background sites since 2010 and was measured at an annual mean concentration of 0.24 ng m^−3^ and a 99th percentile of 4 ng m^−3^ (gaseous and particulate phase) in an intensive campaign in 2018 and 2019^32^. Chlorothalonil was also detected in rainwater as the most concentrated compound in nearly all samples in a campaign performed in an agricultural region of the United States in the late 2000s^33^. It was also observed in rivers, lakes and seas due to agricultural activities and antifouling paints used in boats^34,35^.

The volatilisation of chlorothalonil was first measured in the late 2000s and was evaluated to represent approximately 1% of the applied quantity on the first day and 5% when integrated over a week^36,37^. Several models have been developed to represent chlorothalonil volatilisation following application^36,38,39^. These models compared positively with volatilisation datasets up to a week following application. Noticeably, these datasets usually encompass fewer than 3 measurements per day, not every day, which limits their usefulness in disentangling the competing processes of volatilisation, penetration, and photodegradation^8,37^.

Although these models suggest that chlorothalonil volatilisation may last more than a week, they have never been evaluated over longer durations. There is, therefore, a crucial need for data to evaluate how long chlorothalonil volatilisation can last and how much it may contribute to population and environmental exposure to this pesticide.

Proton transfer mass spectrometry (PTR-MS) has been used for a decade to monitor pesticides, but only indoors^40–42^ or in controlled experiments^43^. The use of PTR-MS for online monitoring outdoor gaseous concentrations of pesticides has never been reported, nor has its use to infer volatilisation from applied crops been published.

In this study, we report for the first time the measurement of chlorothalonil gaseous concentration over a treated wheat field using a highly sensitive proton-transfer-reaction, time-of-flight, quad-injection, mass-spectrometer (PTR-QI-TOF-MS), in a farm typical of the Île-de-France region surrounding Paris, a very productive agricultural area in France. We monitored the concentration at 1.9 m above the surface over three weeks. We computed the volatilisation of gaseous chlorothalonil by inverse dispersion modelling at a 30-minute temporal resolution using a well-established inversion model supplemented with a comprehensive Monte-Carlo-based uncertainty calculation^44–46^. A well-established thermo-desorption, gas-chromatography, mass-spectrometer (TD-GC-MS) was used over the first 3 days of the campaign to calibrate the chlorothalonil concentrations measured with the PTR-Qi-TOF-MS by co-locate sampling. The TD-GC-MS was also used to measure volatilisation by an independent method, the aerodynamic gradient method^37^. It has to be mentioned that the sampling duration for the TD-GC-MS approach lasted from a few hours during the day to all night long. A resistance analogy^47^ was then used to infer the surface concentration and partial pressure of chlorothalonil, which, by comparison with thermodynamic data, allows for the interpretation of the processes underlying the observed volatilisation dynamics. Finally, we computed the cumulated chlorothalonil volatilisation over the campaign duration.

## Results

**Fig. 1:**
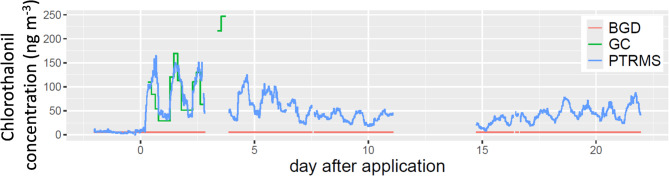
Chlorothalonil concentrations measured using the online PTR-QI-TOF-MS (PTRMS) and the GC‒MS (GC) techniques at 1.9 m above ground at the same location. BGD is the background concentration assumed to be equal to the concentration measured by PTR-Qi-TOF-MS in the field before chlorothalonil application on 17 April.

The chlorothalonil concentration measured hourly using the online PTR-QI-TOF-MS increased sharply, from 5.1 (± 2.4) ng m^−3^ the day before application to 90 (± 40) ng m^−3^ afterwards, revealing a clear emission signal (daily mean ± std. dev.) (Fig. 1**)**. The PTR-QI-TOF-MS and TD-GC‒MS concentration dynamics agreed within the first 3 days. Chlorothalonil concentration showed an expected daily variability, with a maximum around noon (at approximately 14 h UT) and a nonzero minimum at approximately 6 h UT, suggesting temperature-driven emission. The most novel finding of this study is that the concentration remained high for the three weeks of observation, with a daily maximum concentration greater than 50 ng m^−3^ at the end of the observation period. This is the first study in which the chlorothalonil concentration was measured at a temporal resolution of 30 minute for such a long period. The sustained concentration observed over the last 7 days cannot be attributed to an additional source in the surroundings, as confirmed by a footprint analysis showing a maximum 2% contribution from a surrounding field in the north that received chlorothalonil on 29 April (Supp. Figure 1).

### Chlorothalonil volatilisation

**Fig. 2:**
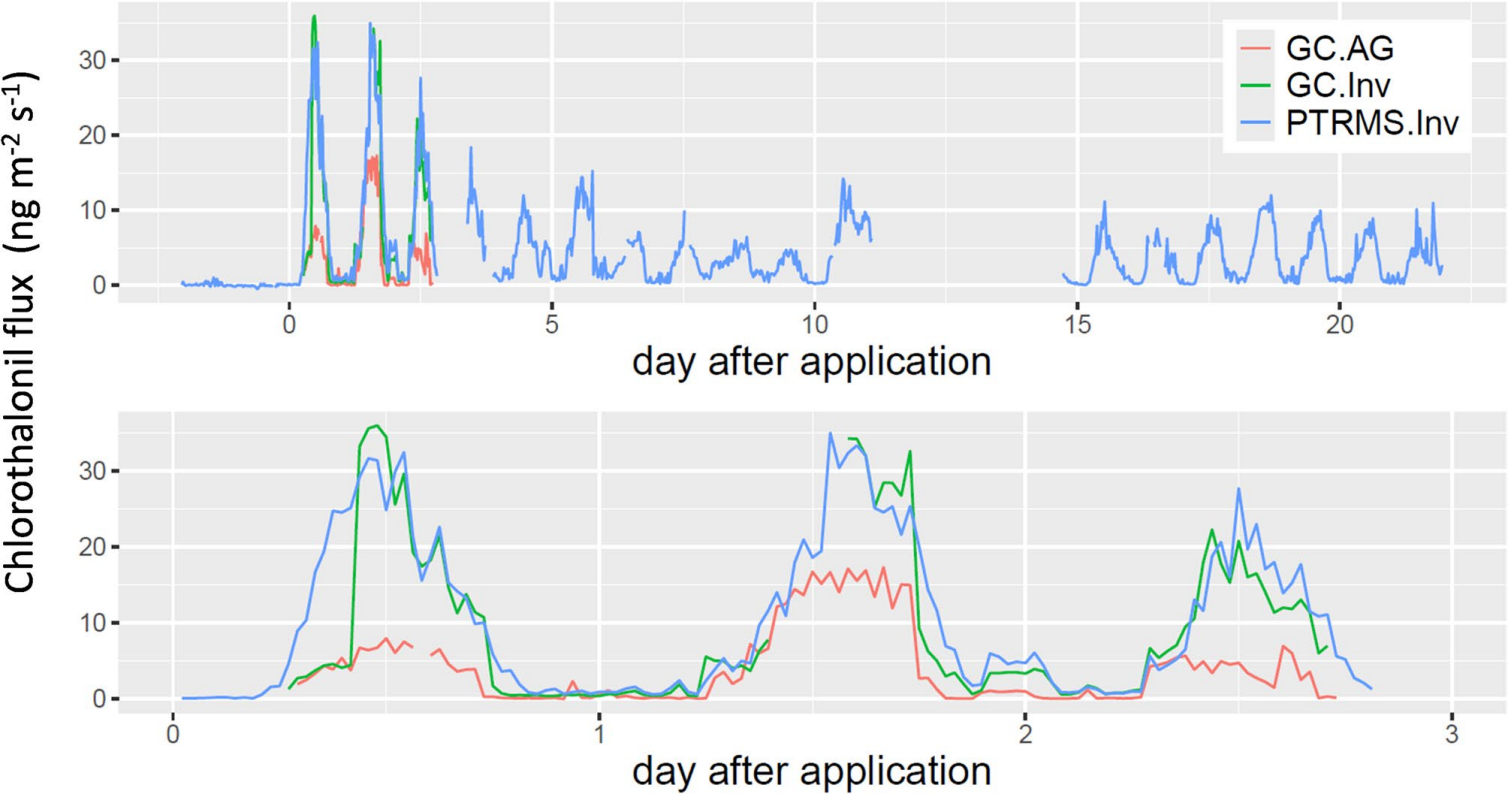
Chlorothalonil flux computed with the PTR-QI-TOF-MS inverse dispersion method (PTRMS.Inv) and the TD-GC‒MS aerodynamic gradient method (GC.AG). The inverse method was also applied at the TD-GC-MS measurements at 1.25 m height (GC.Inv) to control the field’s flux homogeneity. The top panel shows the entire period, and the bottom focuses on the first three days after chlorothalonil application. The discrepancy between the aerodynamic and inversion methods is explained by a zone near the GC mast where no pesticide was applied due to tractor manoeuvres.

Chlorothalonil volatilisation was inferred by atmospheric inversion of the online measured concentrations with the PTR-Qi-TOF-MS and the independent offline TD-GC‒MS aerodynamic gradient method^48^which was only deployed during the first three days of the campaign. The TD-GC-MS aerodynamic gradient method gave smaller fluxes than the inversion method most of the time (Fig. 2 and Supp. Figure 2a). This behaviour was fully explained by the lack of pesticide application around the TD-GC-MS mast, leading to biased vertical gradients when the wind was blowing from that zone (Supp. Figure 2b). When the wind was blowing out from this zone the two methods agreed within the expected range of uncertainty (slope 0.84, R2 0.95)^49^. The flux inferred by atmospheric inversion of the TD-GC-MS was consistent with the PTR-Qi-TOF-MS flux, showing the chlorothalonil flux was homogeneous over the field, except in the application exclusion zone around the GC mast. The chlorothalonil volatilisation showed a burst after application that reached a maximum above 30 ng m^−2^ s^−1^ the following day and a decrease during the next 5 days. We note that volatilisation lasted for three weeks and did not decrease toward the end of the campaign. Indeed, volatilisation was sustained, with a daily maximum of approximately 10 ng m^−2^ s^−1^ during that period, a level that is still 1/3 of the volatilisation rate the day following application. To our knowledge, this is the first reported study in the field or the laboratory showing that chlorothalonil volatilisation lasts more than 10 days. Previous studies have reported only a few measurements per day and not every day^8,37,50^. The 30-minute temporal resolution of the measured flux is also novel, which allowed for a better process understanding, as seen in the next section.

### Interpretation of the chlorothalonil volatilisation dynamics

**Fig. 3:**
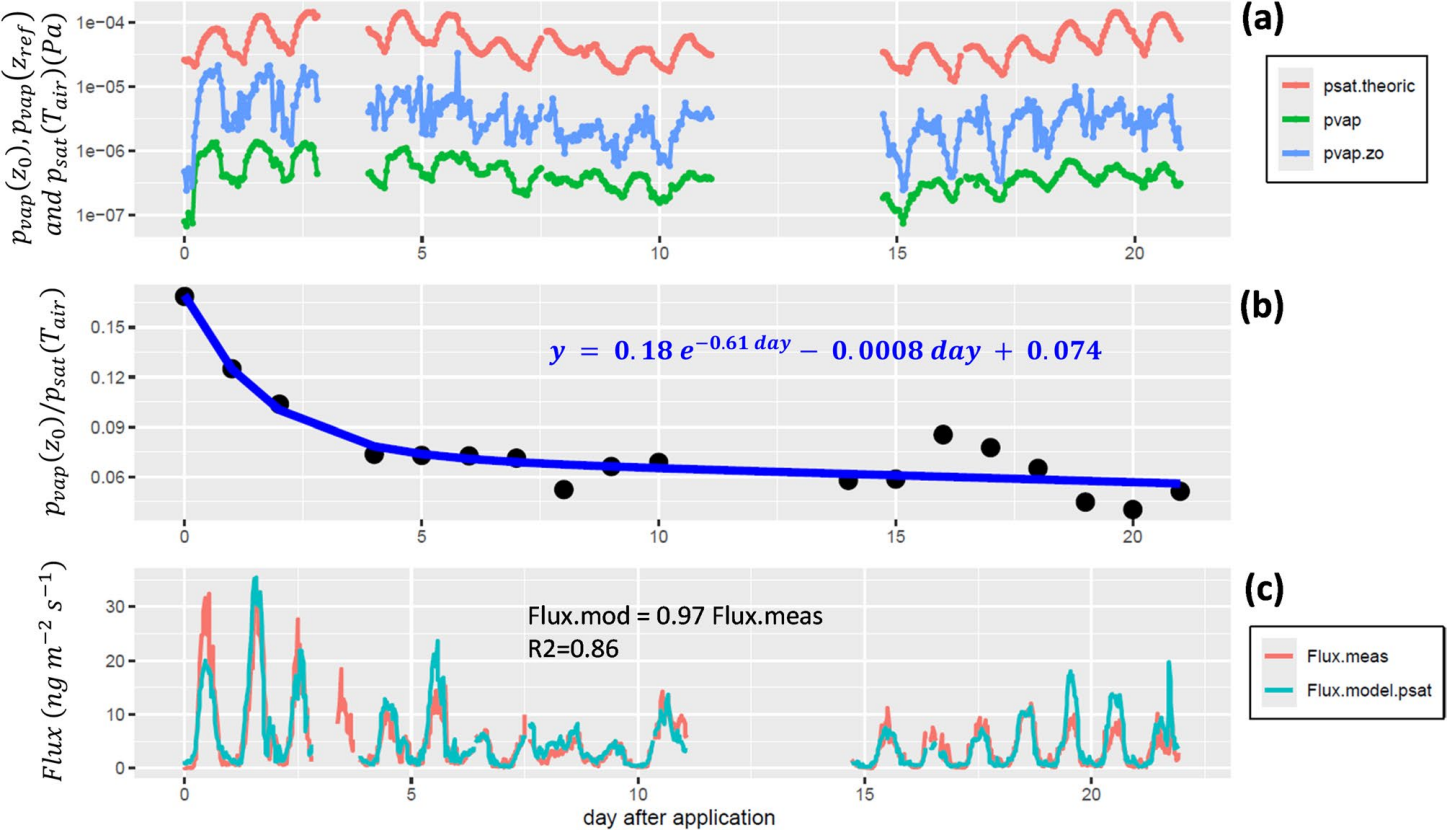
(**a**) Chlorothalonil vapour pressure at the reference height (pvap) and the canopy surface (pvap.zo), and saturation vapour pressure at the air temperature (psat.theoric). (**b**) Ratio of the vapour pressure at the surface *p*_*vap*_(*z*_0_) to the saturation vapour pressure of pure chlorothalonil p_*sat*_(*T*_*air*_) (black points) and the fitted function (bold blue curve). (**c**) Measured and modelled chlorothalonil volatilisation flux. The modelled flux was computed using the blue line function fitted in (**b**) and Eqs. (5–10) (see methods). In (**b**), the standard errors on the equation coefficients are 0.04, 0.2, 0.0006 and 0.01, from left to right.

Acknowledging that transfer in the atmospheric boundary layer is a well-established process^51^, we computed the chlorothalonil surface concentration *C*(*z*_0_) and its corresponding vapour pressure $\:{p}_{vap}\left({z}_{0}\right)$ from measured concentrations, fluxes and transfer resistances (see methods). We observe that the surface pressure $\:{p}_{vap}\left({z}_{0}\right)$ and the pressure at the measurement height $\:{p}_{vap}\left({z}_{ref}\right)$ were both much smaller than the saturation vapour pressure of chlorothalonil $\:{p}_{sat}\left({T}_{air}\right)$ (Fig. 3a), which was expected due to volatilisation. Most interestingly, the ratio $\:r={p}_{vap}\left({z}_{0}\right)/{p}_{sat}\left({T}_{air}\right)$ was around 0.18 just after chlorothalonil application and decreased exponentially at a rate of 0.61 day^−1^ during the first 5 days and then linearly afterwards to reach a minimum of around 0.05 (Fig. 3b). The flux computed using a fitted function of the ratio *r* (Fig. 3b) and Eqs. (5–10) (see methods) agreed remarkably well with the measured flux over the entire period of observation, except for a marked underestimation on the first day and a slight overestimation towards the end of the observation period (Fig. 3c). If we interpret the ratio $\:r$ as the fraction of the canopy and ground surfaces covered by unbound chlorothalonil (which is therefore available for transfer), we can conclude from Fig. 3 that 18% of the crop and soil surface was covered after application and 5% of it was covered three weeks later.

### Cumulated chlorothalonil volatilisation

**Fig. 4:**
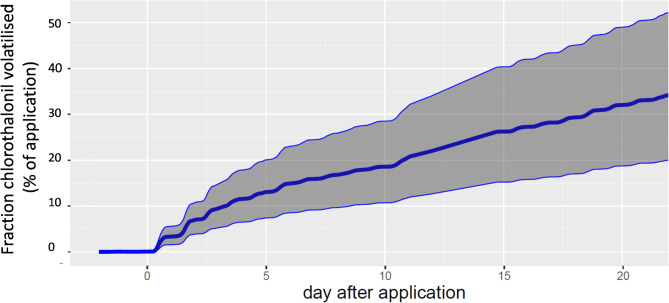
Cumulative volatilisation of chlorothalonil. Mean (blue line), and the 5th-95th percentiles (grey shaded area) evaluated with a Monte-Carlo approach.

Integrating the measured chlorothalonil volatilisation fluxes over the entire period after gap-filling the missing data (see Methods), and computing the uncertainties with a Monte Carlo method, we estimated the range of atmospheric chlorothalonil losses by volatilisation to be between 18% and 48% of the applied dose after three weeks (Fig. 4). After one week, we note that the volatilisation averaged 15% [8% − 23%] of the applied dose, which is in the upper range of volatilisation reported in the literature, amounting to a maximum of 10%^8,36,39,52^.

## Discussion

After application in the field, chlorothalonil volatilisation arises from the combination of thermodynamic equilibrium between the gas, liquid, and solid phases both in the soil and at the plant surface^36^. It is limited by transfer rates to the atmosphere. Volatilisation competes with other dissipation processes, such as photodissociation at the plant surface, transfer into the plant, wash-off, (bio)degradation in the plant and soil, run-off and lixiviation^53^. Chlorothalonil volatilisation dynamics have been well reproduced by models including the PEARL model^36^ and the SurfAtm-Pesticide model^54^. However, validation was performed over a few days only and with a meagre temporal resolution. In this study, we found that chlorothalonil volatilisation can last several weeks.

We further found that the observed chlorothalonil volatilisation dynamics could be reproduced by a big-leaf resistance scheme (see method section and Supp. Figure 3) and surface vapour pressure *p*_vap_(*z*_0_) modelled as a decreasing function *r(time)* of the saturation vapour pressure *p*_sat_(T_a_): *p*_vap_(z_0_) = *r*(time) × *p*_sat_(T_a_) (Fig. 3b and c). We can interpret the ratio *r* as the proportion of the leaf and soil surfaces covered by unbound chlorothalonil, which is available for volatilisation. With this assumption, the observed decrease in *r* can be viewed as a decrease in the pool of chlorothalonil available for volatilisation. This would mean that if the decrease in the pool is faster than the measured volatilisation rate, a dissipation or transfer process other than volatilisation should be active.

**Fig. 5:**
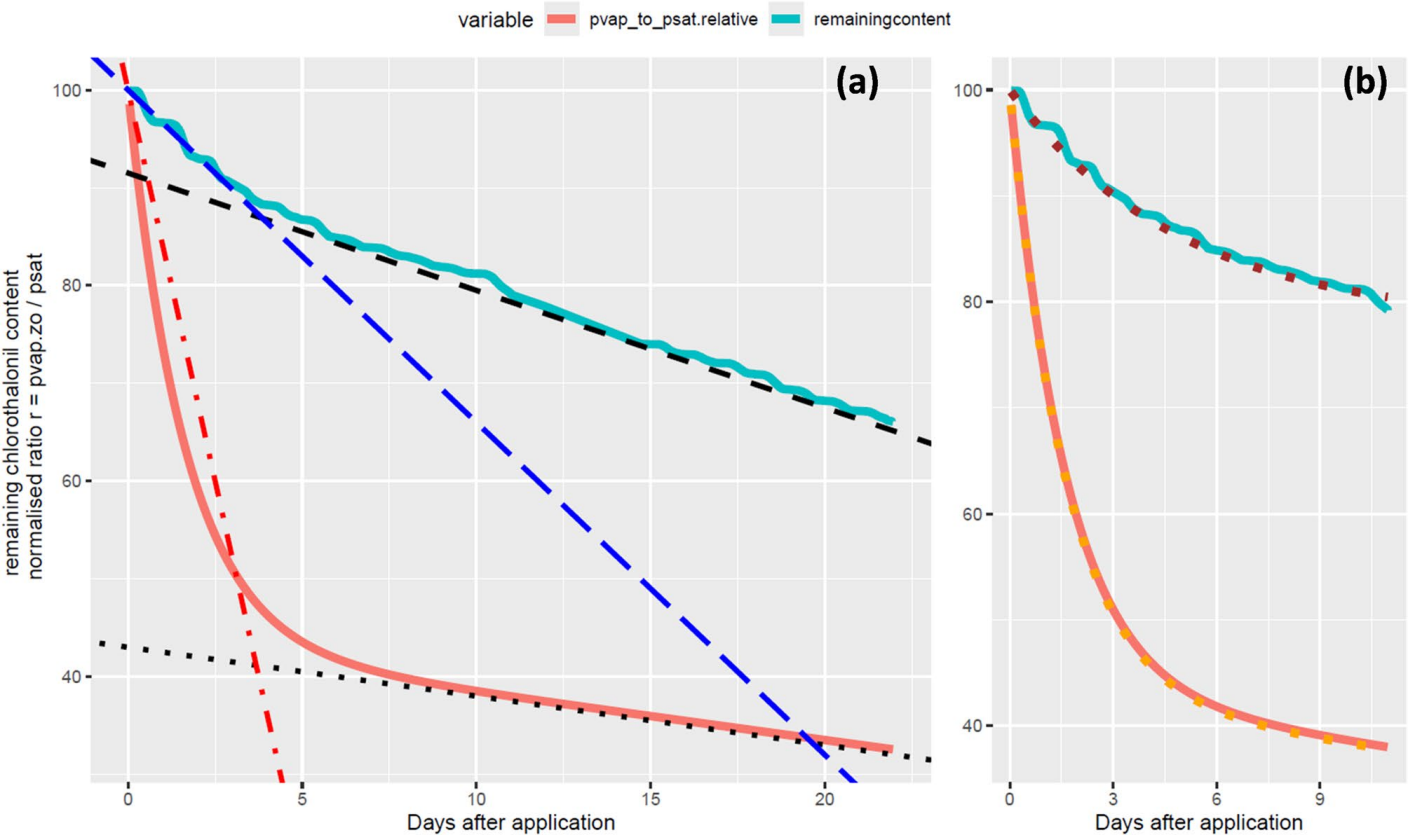
(**a**) Normalised ratio$\:\:r/max\left(r\right)*100$, where $\:r={p}_{vap}\left({z}_{0}\right)/{p}_{sat}\left({T}_{air}\right)$ (bold red line), and remaining chlorothalonil content in the field computed as 100 * (1 – (cumulated chlorothalonil volatilisation)/(application dose)) (bold blue line). The black dash line shows the slope − 1.2%, while the black dotted line shows the slope − 0.5%, which correspond to the asymptotical behaviours of the blue and red curves respectively. The red dash-dotted line shows a slope − 16%, and the blue long-dash line the slope of −3.4%, fitting the start of the red and blue curves, respectively. All these slopes can be interpreted as the percentage decrease per day of the cumulated chlorothalonil volatilisation, and the ratio *r* (which is a proxy of the ecosystem surface covered by chlorothalonil), respectively. (**b**) fittings with double exponential functions of, the initial decrease of the normalised ratio $\:r$ (orange dotted line) with y = 57*exp(−0.61*(days)) + 43*exp(−0.012*(days)), and the remaining chlorothalonil content in the field (brown dotted line) with y = 22*exp(−0.20*(days)) + 78.

This is indeed what we observe during the first 5 days following application, where the decrease in *r* was approximately 16% per day, which is much faster than the 3.4% decrease per day that would have been observed if volatilisation was the only loss process during that period (Fig. 5). This demonstrates that either or both a strong dissipation or transfer process decreased the chlorothalonil availability during that period. As a confirmation, we found that the dissipation first-order constant of *r* during the first days (0.61 ± 0.2 day^−1^, Figs. 3b and 5b) was almost equal to the sum of (a) the photodegradation constant of 0.14 day^−1^^36,55^, (b) the penetration constant of 0.23 day^−1^^39,55^, as both reported in previous works and (c) the volatilisation constant found here (0.20 day^−1^ (Fig. 5b), which sums up to 0.57 day^−1^.

After this first phase, from day 5 to day 22 following application, *r* decreased linearly at 0.5% per day, while a sustained and constant daily volatilisation flux of 1.2% of the initial content was observed during that period (Fig. 5a). This suggests that volatilisation was the dominant dissipation process sustained by the presence of unbounded chlorothalonil at the surface of the leaves or ground during the two latest observational weeks. We can further assume that the dissipation processes observed during the first 5 days following application were no longer effective after day 5. Otherwise, the decrease in *r* would have been faster than the volatilisation flux. On the contrary, we can infer that chlorothalonil is remobilised to sustain volatilisation. The reason for such an observation can only be hypothesised: either or both the chlorothalonil photodegradation or its leaf penetration were reduced. One explanation would be that after 5 days, the chlorothalonil applied on the leaves penetrated the leaves or degraded and was therefore unavailable for further dissipation. We can hypothesise that the volatilisation observed afterwards was coming from a pool of chlorothalonil still available, which should be in a part of the canopy that was not exposed to the sun, so photodegradation should have been negligible. Based on the decrease of *r*, we evaluate that 55% of the applied chlorothalonil would have been dissipated in the 5 first days (Fig. 5). This amount is larger than the amount applied on the leaves as measured with filter exposure (32%, see methods), meaning that part of the dissipation would occur on the pesticide on the ground. Bounding of the pesticide on the organic fraction of the soil is indeed expected^54,56^.

The remobilisation of chlorothalonil for volatilisation during the last two weeks of the study could be explained by mobility in the leaves. Indeed, the current understanding of penetration is that it is unidirectional, assuming that the pesticide is bound to plant tissues once it has penetrated and is hence unavailable for volatilisation^39,50,57,58^. This hypothesis has been, however, contradicted by the findings of several experiments showing polar pesticide mobility inside plants^59^ a feature also observed for triazoles, which have similar polarities as chlorothalonil (similar *K*_ow_, Log Kow = 2.94 for chlorothalonil compared to 2.77, 3.11 and 3.44 for the 3 triazoles)^60^. We can therefore hypothesise that a significant fraction of chlorothalonil that had penetrated the leaves may be unbound and free to volatilise by transfer through either the stomata or the cuticles. Further support for this hypothesis is given by Wang et al.^61^ who recently reported that cuticles of apple and jujube are efficient adsorbents of chlorothalonil but can also desorb around 40% of the previously adsorbed fungicide. Several measurements of fruits^62^ have shown that chlorothalonil was still present in leaf tissues 76 days after application at concentrations a fifth of the initial concentrations, indicating a half-life longer than 30 days in leaves, which is compatible with the volatilisation dynamics observed in our study. Finally, the rain events on 22 and 30 April did not significantly reduce volatilisation, suggesting that leaf washing was not an efficient process for diminishing chlorothalonil volatilisation, as opposed to what models usually consider^36^ further indicating that the chlorothalonil was shed from rain as would happen if it were either inside the leaves or at the ground.

The cumulated quantity of the volatilised fungicide ranged from 20 to 50% of the applied quantity three weeks after application and averaged 30% (Fig. 4). We should also note that this amount is conservative because we stopped measuring before the emissions vanished. Our findings, therefore, show that chlorothalonil volatilisation is a significant route for atmospheric contamination. Other transport routes, such as spray drift deposition occurring during the application, amount to only a few per cent of the applied dose for arable crops^63,64^, while the fraction of spray droplets emitted to the atmosphere is expected to represent only a few per cent of the application dose for boom sprayers^65^. Furthermore, in this study, we measured an application dose at the top of the canopy close to the theoretical applied dose (see methods and Supp. Figure 4), thus confirming that spray drift represented a negligible atmospheric transfer. We therefore deduce that volatilisation was chlorothalonil’s most significant atmospheric contamination route in our study.

Extending our findings at the national level by assuming that our results are representative of France, we calculate that if 30% of the 1700 tons applied in France in 2018 should have been volatilised, that would have led to an amount approximately equal to 510 tons year^−1^ emitted to the atmosphere. Once emitted, chlorothalonil can be transported to long distances or deposited locally. The concentrations measured above the field and in its vicinity are within the range of measured concentrations in similar agricultural regions. These high concentrations suggest that the dry deposition of gaseous chlorothalonil in the surrounding areas of the treated fields could be very significant. Indeed, as a rather water-soluble compound (solubility in water of 0.81 mg/L), gaseous chlorothalonil can be readily dry deposited within hundreds of meters of the application field, as shown by Bedos et al.^66^. This was also reported for other soluble pesticides^55^. Similarly, pesticides may also be removed from the atmosphere by rain to contaminate larger areas, as was reported for soluble pesticides^67–77^. In particular, Huskes and Levsen^69^ found chlorothalonil the most concentrated compound in rainwater sampled in Germany (up to 1.1 µg L^−1^), which gives ground to our findings that a significant fraction of chlorothalonil should be volatilised, leading to a contamination of the atmosphere and as a consequence, high concentrations in the rainwater.

Although chlorothalonil is now banned in Europe, it is still used in 75 countries, as claimed by its supplier Syngenta. Its use in the USA has been relatively constant for 30 years and varies between 3600 and 5400 tons per year^78^. Our study suggested that a significant fraction of this pesticide could be lost to the atmosphere and eventually deposited in rainwater. The high concentration of rainwater found by Potter et al. (2017)^33^ (median of 0.15 µg L^−1^, maximum of 4.5 µg L^−1^) confirms that atmospheric contamination may be a severe issue of chlorothalonil use in the USA. We may also hypothesise that other volatile and soluble pesticides are likely to behave similarly^66^. Therefore, we should expect them to contribute significantly to regional contamination through atmospheric transport and deposition, as Couvidat et al. (2022) suggested.

The combined use of online PTR-QI-TOF-MS and inverse dispersion modelling proves to be a robust approach for detecting gaseous pesticides in the atmosphere and estimating their volatilisation from treated fields. Our study indicates that this approach allows for a much better determination of the actual volatilisation rates of some pesticides over long periods and their potential spread over larger scales. Such datasets are urgently needed to explain the observed contamination of the atmosphere and rainwater by pesticides^73,74,79,80^.

## Methods

### Field site and pesticide application

The experiment was conducted in a 20-ha winter wheat field on a mixed farm (with grains, milk and meat production) in the Parisian region of France (field A in Supp. Figure 5). Field A is an ecosystem site (labelled FR-Gri) of the Integrated Carbon Observation System (ICOS) European Research Infrastructure Consortium, meaning that meteorological, heat and carbon fluxes were measured with the highest standard methods. At the time of the experiment, the wheat plants were at the elongation stage, with a mean height of 0.5 m, a leaf area index of 3 m^2^ m^−2,^ and a total dry weight of 300 kg m^−2^. The field was treated with a mixture of three fungicides (Cherokee), namely, cyproconazole (50 g L^−1^), propiconazole (62.5 g L^−1^), and chlorothalonil (375 g L^−1^), on 17 April 2018 to protect against visible fungal development on the crop that had started a few days previously. The chlorothalonil target dose was 487 g ha^−1^ of the active substance. The same fungicide was applied in adjacent wheat fields during the experimental period: Field B and Field C received chlorothalonil on the same day as Field A, while Field D received it on 29 April (Supp. Figure 5). Fields B and C were therefore considered a possible source, while field D was proved too far away to generate significant concentrations at the field site. The same dose was applied to all the fields except Field B, which received 68% of that dose. Fungicide application was performed together with a growth regulator (Medaxtop) containing mepiquat-chlorure (300 g L^−1^) and prohexadione-calcium (50 g L-1) at a target dose between 0.25 and 0.35 L ha^−1^.

Turbulence statistics, wind direction (WD), friction velocity (u_*_), and Obukhov length (*L*) were measured with two 3-dimensional ultrasonic anemometers placed at the sampling locations of the PTR-QI-TOF-MS (R3-50, GILL, UK) and the TD-GC‒MS (HS-50, GILL, UK). Anemometers and meteorological measurements were recorded every 30 min and quality-checked according to the ICOS standard^81^ (Supp. Figure 6).

### Chlorothalonil application dose measurement

The application dose was calculated with two methods. (a) The chlorothalonil concentration measured in the application tank before the application was multiplied by the volume of preparation applied and divided by the surface applied. (b) 18 paper filters (9 cm diameter), separated into 3 sets of 6, were placed just above the canopy top at three locations in the field. The applied dose was calculated as the chlorothalonil content measured in the filters divided by the filters’ area (See Supp. Section B). A set of 18 additional filters was placed at the ground grouped at the same locations to evaluate the proportion of the fungicide reaching the ground.

The filters were collected just after exposure to minimize volatilisation losses (< 10 min). They were polled by 2 and put in 250 mL amber flasks using tweezers. 200 mL of ethyl acetate was then added to the flask just after filter collection. The flasks were agitated overnight to extract the pesticide. A 2 mL sample from each extraction flask was then transferred into an amber vial and stored at 4 °C prior to analysis. The chlorothalonil concentrations in the extraction solutions were subsequently quantified via GC‒MS (see TD-GC‒MS analysis section for the detail on the GC-MS).

The measured application dose was 544 ± 59 g ha^−1^ according to the method (a) and 540 ± 54 g ha^−1^ according to the method (b) (Supp. Table1). A Student t-test further showed that the two estimations were not significantly different (t = 0.11, df = 5.4, p-value = 0.91). This agreement gives great confidence that volatilisation did not significantly affect the filters’ pesticide content. It further gives confidence in the estimation of the real applied dose, which was 11% larger than the farmer target’s dose. The filters placed at the ground received 370 ± 37 g ha^−1^, which represented 68% of the applied dose (Supp. Section B and Supp. Figure 4). The difference between the applied dose and the dose at the ground (32%) was considered as deposited onto the vegetation.

### Chlorothalonil atmospheric concentration measurement

A PTR-QI-TOF-MS (online analysis) was installed in a temperature-controlled field laboratory located between fields A and B. Air was sampled with a pump (Bush SECO SV1010) at a flow rate of 50 NL min^−1^ controlled with a mass flow meter (Bronkhorst) from a 1.9 m height mast with a 3/8-inch internal diameter PFA Teflon line insulated from air and sun and heated at 60 °C. The mast was installed 40 m deep inside wheat field A (Supp. Figure 7). The air was then subsampled from the 3/8-inch tube in a 1/16-inch PFA tube into the PTRMS at 500 mL min^−1^.

A separate gradient mast was installed in the middle of the wheat field to estimate the chlorothalonil flux above the canopy with a TD-GC-MS method. Three Tenax TA adsorbent sampling cartridges were set up at 0.63, 1.23, and 2.03 m above the ground to measure the pesticide concentration in the air. Ambient air was sampled for 3 to 12 h at 0.5 L min^−1^ using a pump and a mass flow controller to regulate the flow rate (Bronkhorst EL-Flow). Chlorothalonil air concentrations from the cartridges were later determined by thermodesorption (TD), and GC‒MS (see next section for details). Additional cartridges were collocated with the PTR-QI-TOF-MS sampling point for calibration purposes. After exposure, all cartridges were stored in the dark at 4 °C until analysis. Cartridge measurements were performed just after pesticide application over three days from 17 to 19 April.

### TD-GC‒MS analysis

Tenax tubes were analysed by TD-GC-MS, as detailed in Decuq et al. (2022)^82^. Tubes were desorbed using a Thermo Desorption Unit (Gerstel, DE) with a 10 min 100 °C min^−1^ ramp from 50 to 290 °C and a 100 mL min^−1^ helium carrier flow, followed by a cryo-focus in a temperature vaporisation injector at −20 °C using a baffled glass liner. Compound separation was carried out using an Agilent 7890B gas chromatograph equipped with a capillary column (30 m length, 0.25 mm internal diameter, 0.25 μm df, HP-5MSUI column, Agilent) with a 20 °C min^−1^ ramp from 90 °C to 300 °C, a 1 min holding time, and a 1.5 mL min^−1^ helium carrier flow. The GC‒MS instrument was used with an electronic impact mode set to + 70 eV, a SCAN mode with a 30 to 380 m/z range, and a SIM (*Selected Ion Monitoring*) mode with a target ion 266 m/z for chlorothalonil. The ion source temperature was set to 230 °C, and the quadrupole temperature was set to 150 °C. TD-GC‒MS data were processed with MassHunter software version B.07.04.5560 (Agilent Technologies, Inc.). For filter and applied solution extraction quantification (dose estimation), because liquid solution analysis was required here, TDU was not used and replaced by the PTV injector used in solvent vent mode. 1 µl of extraction solution was injected at a split ratio of 1/10.

To ensure accurate quantification of both filters’ extraction solution and thermo-desorbed Tenax tubes, we used SIM (Selected Ion Monitoring) chromatograms. All chlorothalonil chromatographic peaks were checked one by one by analysing (a) the retention time of the peak, (b) the presence of chlorothalonil-specific ions. If during chromatogram control any doubt existed as to the presence of interference (peak broadening, for example), we also used deconvolution (Unknowns Analysis (B.06.00 software) to validate that no interference remained. Deconvolution was only used for 4 samples out of around 150 samples. See Supp. Section C for example Chromatograms.

Standard solutions with concentrations ranging from 2.5 to 120 ng µL^−1^ were prepared for quantification by diluting a commercial salt of chlorothalonil (CIL, Cluzeau, FR) in ethyl acetate (Carlo Erba). Clean Tenax tubes were spiked with 1 µL of standard solution, taken up with a glass syringe, and deposited on the grid of the Tenax tube. The tubes were then dried to evaporate the ethyl acetate solvent with helium at 0.5 ± 0.05 L min^−1^ for 15 min and used as a standard for quantification.

The TD extraction efficiency was determined in the laboratory prior to the experiment: Tenax tubes were doped with known chlorothalonil quantities following the same protocol as described above for standard solution. Two thermo-desorption cycles were then performed on each cartridge to determine the extraction efficiency. No detectable concentration was measured during the second extraction, hence confirming that TD extracted all the chlorothalonil adsorbed in the Tenax tube during the first extraction cycle. To further control the Tenax tube adsorption efficiency in the field, a second Tenax tube was placed after the exposed one during the first sampling period (highest concentrations), and during the nights (the longest exposure period). No detectable chlorothalonil concentration was measured in the second tube during this study, further confirming that the exposed tube trapped all the chlorothalonil in the sampled air.

Finally, to ensure the trueness of the quantification method, we used reference standards to produce our standard solutions but also as quality control for our calibration curve. Standard solutions were regularly analysed during the analysis step, allowing for correction of any analysis drift.

### PTR-QI-TOF-MS field setup and concentration calculations

The PTR-QI-TOF-MS (Ionicon, AU), sampled air continuously at 50 mL min^−1^ into a drift tube where gases with a proton affinity larger than that of water vapour get protonated by transfer from H_3_O^+^ produced in a highly controlled ion source. The ions are then guided into a high vacuum, time of flight detector with a combination of a quad injection system and electromagnetic lenses. The PTR-QI-TOF-MS was operated with a drift pressure of 3.5 mbar, a drift temperature of 80 °C and a drift voltage of 878 V. These conditions were set to ensure a constant E/N ratio (where E is the electric field strength and N is the gas number density) of approximately 133 Td (1 Td = 10^−17^ V cm^−2^), which limited fragmentation and the sensitivity of the protonation rate to variations in relative humidity^83,84^.

The PTR-QI-TOF-MS was set up on 14 April and set off on 10 May. Daily, the instrument background signal was controlled using high-purity zero air (Alphagaz 1, zero air: 80% nitrogen, 20% oxygen, purity: 99.9999%, Air Liquide). Two interruptions in the measurements occurred on 20 April and from 28 April to 1 May due to an electrical power breakdown.

**Fig. 6:**
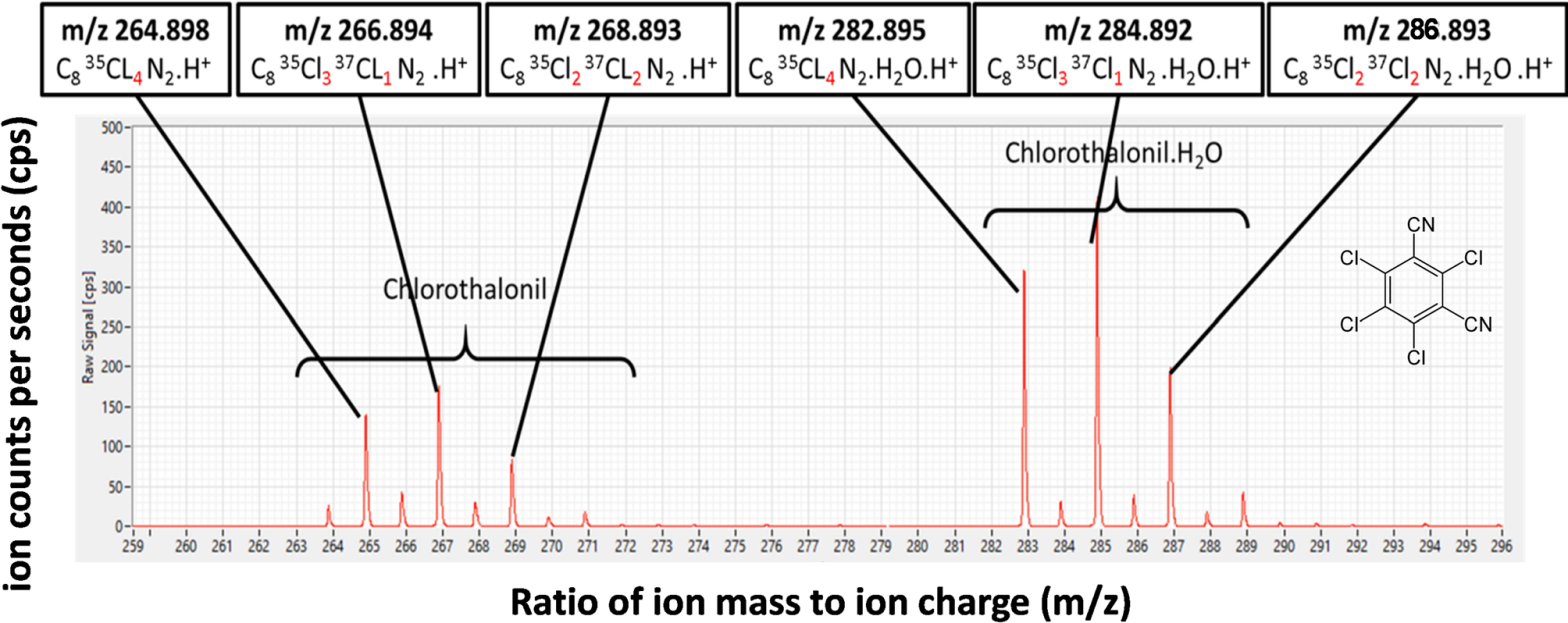
Example mass spectrum of chlorothalonil measured with the PTR-QI-TOF-MS. Air was sampled from a flask in the laboratory. Six-ion pics showing the protonated isotopes (hydrated and not hydrated) are shown.

Prior to field measurements, the chlorothalonil mass spectrum was determined in the laboratory using a heated flask containing chlorothalonil flushed with ultra-purified air. Two isotopic massifs with protonated ions corresponding to the three significant isotopes of C_8_Cl_4_N_2_ at approximately m/z 265 and corresponding clusters with bound water molecules at approximately m/z 283 were observed (Fig. 6). The ion counts per second (cps) for chlorothalonil was computed by summing all 6 ion peaks measured on the mass spectrometer detector. The cps was used to calculate the “uncalibrated” chlorothalonil concentration following Loubet et al.. (2022)^85^.

The chlorothalonil concentrations measured by PTR-Qi-TOF-MS were subsequently calibrated against those measured with TD-GC‒MS at the exact location during the first 3 days of the experiment (Supp. Figure 8). The scatter on the calibration slope (from 1.07 to 1.18) was used to compute the uncertainty in the PTR-QI-TOF-MS concentration evaluated as ± 14% (1σ). This relative uncertainty was used to determine the uncertainty of the chlorothalonil concentration in the rest of the study. Supp. Figure 8 was also used to compute the sensitivity of the PTR-Qi-TOF-MS for chlorothalonil, which was found to be 0.7 cps (ng m^−3^)^−1^, or 8 cps ppt^−1^. Since it is necessary to have at least 1 cps to detect an ion over a second, the limit of detection (LOD) at a sampling rate of 1 s can hence be estimated to be ~ 1 ng m^−3^, and therefore 0.0005 ng m^−3^ for 30 min averaging. An alternative way to estimate the detection limit was to measure the cps signal for zero air at m/z = 285, which was measured around 0.1 cps for 30 min averaging, which gives a limit of detection for chlorothalonil around ~ 0.1 ng m^−3^ over 30 min. This latter LOD estimate is much larger than the 0.0005 ng m-3 estimation based on sensitivity but is more realistic as it includes the instrument noise. We therefore retained an LOD of 0.1 ng m^−3^.

### Inference of chlorothalonil volatilisation by atmospheric inversion

We inferred chlorothalonil volatilisation using a well-established inverse dispersion method^44,45^ that was already successfully compared to independent volatilisation flux measurement for chlorothalonil and fenpropidine^37^. The method consists in retrieval of the volatilisation flux (*F* in µg m^−2^ s^−1^) from a delimited area based on measured concentration above this area.

In brief, the method requires to know the background concentration ($\:{C}_{bgd}$ in µg m^−3^), which is the concentration upwind of the source, and to measure the concentration ($\:C$ in µg m^−3^) above the source area at a location $\:\left({{x}_{ref},z}_{ref}\right)$. Assuming a source strength *F* in the source area, and no chemical sources and sinks in the atmosphere, the superposition principle^86^ stipulates that the concentration at the target point *C* can be expressed as:

1 $$\:C=D\times\:F+{C}_{bgd}$$

Where $\:D$ is the dispersion coefficient (s m^−1^) that expresses the influence of the source *F* to the concentration at the target location^87^. $\:D$ can be computed with any dispersion model adapted to short range dispersion, including backward Lagrangian stochastic models^88^and semi-analytical solutions of the advection diffusion Eq^89^. Once $\:D$ is modelled, Eq. (1) can be used to determine $\:F$ knowing $\:C$ and $\:{C}_{bgd}$:

2 $$\:F={D}^{-1}\times\:\left(C-{C}_{bgd}\right)$$

Note that $\:{h=D}^{-1}$ (m s^−1^) is also called the concentration footprint, and can be viewed as a transfer coefficient^45^. In this study we used the FIDES model to compute $\:D$ and $\:h$. The FIDES model uses a semi-analytical solution of the advection-diffusion equation implemented in R^44,45,90^. This approach is well established^91^ and was tuned against the backward Lagrangian Stochastic model WindTrax^88^ to account for slight discrepancies in the stability response functions (see Supp. sections s4.2 and s4.3 of Loubet et al. 2018 ^45^). Unlike fully Gaussian models, FIDES assumes power-law vertical profiles of wind speed and turbulent diffusivity, making it appropriate for atmospheric surface layer and inversion problems at heights of a few meters. The details of the FIDES dispersion model equations are given in the Supp. section A.

In Eq. (2), $\:D$ that was modelled with FIDES every 30 min, and the $\:C$ was taken as the concentration measured with PTR-QI-TOF-MS above the source (see maps at Supp. Figure 5 and setup scheme at Supp. Figure 7). The $\:{C}_{bgd}$ was estimated as the average ambient concentration measured during the day before chlorothalonil application. $\:{C}_{bgd}$ was more than an order of magnitude lower than that of *C* after application. Hourly and daily variations in $\:{C}_{bgd}$ were therefore neglected as they would not induce large changes in the source *S*.

The source area was set to fields A, B and C taken all together since all fields were applied with chlorothalonil the same day (Supp. Figure 5). A two-source scenario was computed to evaluate the possible contribution of field D to the inferred flux. To that prospect, the contributions of the ABC and D fields to concentration were estimated separately and compared (Supp. Figure 1). The proportion of each source to the modelled concentration at the PTR-QI-TOF-MS location was computed to evaluate whether a significant contribution originated from field D, which was found to be lower than 2% and, therefore, neglected.

The inverse dispersion method was also applied using the concentration measured at the TD-GC‒MS mast location at the three measurement heights to test the consistency of the inversion method with different sampling locations and analytical techniques (Fig. 5). To do that, in Eq. (2) the concentration measured at the GC-MS location was used and the dispersion coefficient *D* was computed for the TD-GC-MS mast location. The volatilisation flux *F* estimated by the inverse dispersion method using TD-GC‒MS concentrations strongly agreed with the one obtained using the PTR-QI-TOF-MS concentrations (0.88 regression slope and R2 = 0.88; Supp. Figure 2a and 2b left).

### Chlorothalonil volatilisation uncertainty computation

A Monte Carlo approach was used to quantify the uncertainty in the volatilisation flux$\:\:F$. All input data and parameters of the FIDES model, as well as $\:C$ and $\:{C}_{bgd}$ were assigned a Gaussian distribution with a mean and a standard deviation, either measured in the field or set from the literature and reported in Table 1. All the data and parameters were considered uncorrelated with each other. The FIDES model was run 100 times, at each time step, with a combination of each parameter sampled from the multidimensional parameter distribution to provide a distribution of $\:h$, $\:C$ and $\:{C}_{bgd}$, and ultimately compute $\:F$ with Eq. (2).

**Table 1:** Parameter of the FIDES inverse dispersion model used to compute the chlorothalonil flux *F* and its uncertainty with Eq. (2). The Monte Carlo method used each parameter’s mean and standard deviation to quantify the uncertainty in the flux F.

Parameter	Units	Mean	SD	References
Roughness length z_0_	m	0.0035^*^	0.0004^*^	Measured log-normal distribution^49^
Displacement height	m	0.15–0.44^#^	0.03	Measured normal distribution^49^
Sensor location (x, y,z)	(m, m, m)	variable	(1,1,0.1)	GPS positioning error in x and y and soil roughness in z
Friction velocity u_*_	m s^−1^	measured	14% ^$^	Spatial variability of u_*_ as reported by^92^
Wind direction	deg/N	Variable	12.5 deg	Measured spatial standard deviation among 3 sensors
Obukhov length L	-	Variable	40% ^$^	Computed assuming a 14% error on both u_*_ and H
σ_w_^£^	m s^−1^	Variable	14% ^$^	Assumed similar in magnitude to u_*_
$$\:C$$	ng m^−3^	Variable	14% ^$^	Uncertainty based on calibration curve (Supp. Figure 8)
$$\:{C}_{bgd}$$	ng m^−3^	5.7	2.2	Concentration measured during 2 days before application

^*^ log-normal distribution parameters (mean and variance). # varying value taken as $$\:\frac{2}{3}{h}_{c}$$, where $$\:{h}_{c}$$ is the canopy height. $ percentage of the mean value. £ σ_w_ is the lateral wind component standard deviation.

### Chlorothalonil volatilisation computed by the aerodynamic gradient method

The fluxes inferred by inversion were compared during the first 3 days of the campaign to those estimated by an independent method, the GC‒MS aerodynamic gradient method. According to the well-established Monin and Obukhov theory of the atmospheric surface layer, the flux between the surface and the atmosphere is inversely proportional to the vertical concentration gradient^48^. The concentration gradient was measured with TD-GC‒MS Tenax TA tubes at three heights, chosen to be within the turbulent boundary layer estimated with the standard micrometeorological rule of 1/10th of the fetch (distance to the field edge) which averaged 209 m during the measuring period^93,94^. The flux was estimated following the long-established method, e.g.^49^:

3 $$\:F=-{u}_{\text{*}}{C}_{\text{*}}$$

4 $$\:{C}_{\text{*}}=k\frac{\partial\:C}{\partial\:\left(\text{ln}\left(z-d\right)-{{\Psi\:}}_{H}\right)}$$

where $\:C$ is the chlorothalonil concentration, *z* is the height above ground, *d* is the zero-plane displacement, which represents the shift in the aerodynamic “ground” because of the presence of a plant canopy, *k* is von Karman’s constant (*k* = 0.4), and Ψ_H_ is the integrated stability correction function for scalars. Ψ_H_ was calculated from the Obukhov length according to Sutton et al.^95^. The displacement height *d* was determined as 2/3 of the canopy height *h*_c_ and was verified based on the wind speed gradient in this field.

The flux measured with the aerodynamic gradient method was 60% lower than the PTR-QI-TOF-MS inverse flux. This difference could be explained by an application exclusion zone around the GC mast required for the tractor to avoid the nearby ICOS mast (**Supp. Figure 9**). This effect is clearly shown in Supp. Figure 2b-right, where we see that wind sectors from 90 deg/N to 210 deg/N show a lower estimate from the gradient method while the 60 deg/N sector is much closer. Correcting this effect by footprint attribution led to an agreement within 40% (slope = 0.6, R2 = 0.73) of the two methods (Fig. 2).

### Inference of chlorothalonil surface vapour pressure

To evaluate the plausibility of the measured chlorothalonil fluxes and better understand the volatilisation process, it is interesting to compute its surface vapour pressure in the canopy and compare it to the saturation vapour pressure at the same temperature, which is the chlorothalonil vapour pressure expected above a surface saturated with chlorothalonil. Indeed, the ratio between the chlorothalonil surface vapour pressure and the saturation vapour pressure at the leaf surfaces indicates the leaf and soil surface coverage by unbound chlorothalonil.

To retrieve the chlorothalonil surface vapour pressure, we first use a well-established resistance analogue scheme of surface-atmosphere pollutant exchange^47^ to compute the concentration at the leaf surface $\:C\left({z}_{0}\right)$ (Supp. Figure 9). In a resistance scheme, the flux is equal to the concentration difference between leaf surfaces $\:C\left({z}_{0}\right)$ and a reference height $\:C\left({z}_{ref}\right)$, divided by the transfer resistance between these endpoints, which sums up a quasi-boundary layer canopy resistance $\:{R}_{b}$ and an aerodynamic resistance $\:{R}_{a}\left({z}_{ref}\right)$, giving:

5 $$\:F=\frac{C\left({z}_{0}\right)\:-\:C\left({z}_{ref}\right)}{{R}_{b}+{R}_{a}\left({z}_{ref}\right)\:}$$

Here, $\:C\left({z}_{ref}\right)$ is the chlorothalonil concentration measured at *z*_ref_, *F* is the measured flux at the same height, as in Eq. (2), and $\:C\left({z}_{0}\right)$ is the concentration at the leaf surfaces. Simple manipulation of (4) leads to an estimation of $\:C\left({z}_{0}\right)$:

6 $$\:C\left({z}_{0}\right)\:=\:C\left({z}_{ref}\right)+F\times\:\left({R}_{b}+{R}_{a}\left({z}_{ref}\right)\right)$$

In Eq. (6), $\:C\left({z}_{ref}\right)$ and $\:F$ are measured quantities, while the resistances $\:{R}_{b}$ and $\:{R}_{a}\left({z}_{ref}\right)$ were computed based on micrometeorological measurements following Personne et al.^56^:

7 $$\:{R}_{a}\left({z}_{ref}\right)=\frac{\text{log}\left(\frac{{z}_{ref}-d}{{z}_{0}}\right)-{{\Psi\:}}_{H}}{k{u}_{\text{*}}}$$

8 $$\:{R}_{b}=\frac{2}{k{u}_{\text{*}}}{\left(\frac{Sc}{Pr}\:\right)}^{2/3}$$

where *Sc* is the Schmidt number depending on the chlorothalonil diffusivity and Pr is the Prandtl number, and other terms have already been explained. $\:{R}_{a}\left({z}_{ref}\right)$ varied between 10 and 100 s m^−1^ while $\:{R}_{b}$ varied from 20 to 400 s m^−1^ (Supp. Figure 10). From the concentration, one can then compute the chlorothalonil vapour pressure *p*_vap_ (Pa) at both heights *z*_0_ and *z*_ref_ using the ideal gas law:

9 $$\:{p}_{vap}=C\times\:{10}^{-9}\times\:\frac{R\:{(T}_{air}+273.15)\:}{{M}_{Chl}}$$

where *R* is the ideal gas constant (8.14 J K^−1^ m^−1^), $\:{M}_{Chl}$ is the chlorothalonil molar mass (265.9 g mol^−1^), and the 10^−9^ term converts ng to g. The estimated $\:{p}_{vap}\left({z}_{0}\right)$ can then be compared to the chlorothalonil saturated vapor pressure $\:{p}_{sat}\left({T}_{air}\right)$, which is the vapor pressure expected above pure chlorothalonil and was computed from the Clausius–Clapeyron equation and a saturation vapor pressure of $\:{7.6\:10}^{-5}$ Pa at $\:{T}_{ref}=25$, taken from Lichiheb et al.^39^:

10 $$\:{p}_{sat}\left({T}_{air}\right)={7.6\:10}^{-5}{e}^{\frac{95000}{8.3\:}\times\:\left(\frac{1}{\left(25+273.15\right)}-\frac{1}{\left({T}_{air}+273.15\right)}\right)}$$

Equations (9) and (10) were used to compute $\:{p}_{sat}$ and $\:{p}_{vap}\left({z}_{0}\right)$ and their ratio $\:r={p}_{vap}\left({z}_{0}\right)/{p}_{sat}\left({T}_{air}\right)$. Note that we used $\:{T}_{air}$ to compute the vapour pressures as we found it more representative of the whole ecosystem temperature which is in between the canopy top temperature, being exposed to sun during the day and radiative cooling during the night, and the soil temperature that is shed.

### Cumulated chlorothalonil volatilisation

The cumulated chlorothalonil volatilisation was computed as the integral of the volatilisation flux F over time. The gaps in the emission dataset, due to PTR-QI-TOF-MS malfunction, were first gap-filled using Eq. (5) with a parameterised C(z_0_) based on the fitted vapour pressure function shown in Fig. 3a. The resulting modelled flux (Fig. 3b) was then linearly fitted to the measured volatilisation over a 7-day moving window to fill the gaps during the measurement campaign.

## Supplementary Information

Below is the link to the electronic supplementary material.

Supplementary Material 1

## Data Availability

The data and R scripts can be downloaded from the following: Loubet, Benjamin, 2023, “AgriMultiPol Cholortalonil 2018”, https://doi.org/10.15454/QS6ROA.
