# Supplementary material for: First online field measurements of chlorothalonil volatilisation using proton transfer mass spectrometry and inverse modelling
Source: Sci Rep. 2025 Aug 19;15:30426. doi: 10.1038/s41598-025-13898-0 (PMC12365159; doi:10.1038/s41598-025-13898-0)
Supplement: Supplementary file 1 — Supplementary Material 1 [file 41598_2025_13898_MOESM1_ESM.pdf]

First online field measurements of chlorothalonil volatilisation using proton transfer mass spectrometry and inverse modelling
–
Supplementary material

Supplementary section A: details of the FIDES dispersion model

In the FIDES model, the transfer function *D* was estimated by first translating and rotating the x-y plan to set the wind direction *WD* to 0 (align the *x*-axis with the wind vector. This was done by setting the following coordinate transformation $X = (x - x_s)\sin(WD) - (y - y_s)\cos(WD)$, and $Y = (x - x_s)\cos(WD) - (y - y_s)\sin(WD)$, where x, y stands for the target location and x_s, y_s for the source location. All heights are considered as heights above displacement height *d:* $Z = z - d$. In such conditions, the Philip (1959) semi-analytical solution of the advection-diffusion equation reads:

$$U(Z) = aZ^p \quad \text{(S1)}$$

$$K_z(Z) = bZ^n \quad \text{(S2)}$$

$$\sigma_y = \frac{1}{\sqrt{2}} C_y X^{\frac{2-m}{2}} \quad \text{(S3)}$$

$$D(X, Y, Z, Z_s, t) = \frac{1}{\sqrt{2\pi}\sigma_y} \exp\left(-\frac{Y^2}{2\sigma_y^2}\right) \times \frac{(ZZ_s)^{\frac{1-n}{2}}}{b\,\alpha\,X} \times \exp\left(-a\frac{Z^\alpha + Z_s^\alpha}{b\alpha^2 X}\right) \times I_{-\vartheta}\left(\frac{2a(ZZ_s)^{\frac{\alpha}{2}}}{b\alpha^2 X}\right) \quad \text{(S4)}$$

Where *U* is the wind speed, K_z is the vertical diffusivity, $\alpha = 2 + p + n$, $\nu = (1 - n) / \alpha$, and $I_{-\nu}$ is the modified Bessel function of the first kind of order $-\nu$, and Cy and *m* were taken from Sutton (1932). The values of *a*, *b*, *p* and *n* were inferred by linear regression between ln(*U*), ln(K_z) and ln(*Z*), over the height range $2 \times z_0$ to 20 m, using *U*(z) and K_z(z) estimated from the Monin-Obukhov similarity theory as $K_z(Z) = ku_* Z[Sc\emptyset_H(Z/L)]^{-1}$. Here $\emptyset_H(Z/L)$ is the universal stability correction function as in Kaimal and Finnigan (1994), which is $\emptyset_H(Z/L) = (1 + 5.2\,Z/L)$ for $Z/L \geq 0$ and $\emptyset_H(Z/L) = (1 - 16\,Z/L)^{0.5}$ for $Z/L \leq 0$. Following Loubet et al. (2001), to ensure **Eq. (S4)** exists, the source height is taken as $Z_s = 1.01\, z_0$. Note that in FIDES, the stability response functions $\emptyset_H(Z/L)$ were tuned against a Lagrangian Stochastic model as explicated in Loubet et al. (Loubet et al., 2018).

Using **Eq. (S4)**, the dispersion factor of the whole source area S is computed by integrating D over the source area. In practice, x_s and y_s are moved around a grid of 1 m width covering the source area and each value of D computed with eq. (S4) are summed up to provide the D_S.

Supplementary section B: Computation of the application dose

The applied dose was estimated using paper filters placed just above the canopy during application. The applied dose $D_{applied}^{\text{filter}}$ (g ha^{-1}) was computed as the chlorothalonil content measured in the filters divided by the filters' area:

$$D_{applied}^{\text{filter}} = \frac{C_{pest}^{\text{filter}} \times V_{solvent}}{N_{filter} \times F_{area}} \times 1\text{e}^{-5} \quad \text{(S5)}$$

where C_{pest}^{filter} is pesticide concentration measured in the extraction solution (ng µL^{-1}), $V_{solvent}$ the extraction solvent volume put in the flask (200 000 µL), N_{filter} the number of filters pooled by flask (2), F_{area} is the filter area (0.0632 m^{2}). The factor 1e^{-5} is used to obtain g ha^{-1} from ng m^{-2}.

We also evaluated the application dose from the measured concentration in the application tank $D_{applied}^{\text{tank}}$multiplied by the volumetric application rate (L ha^{-1}) as:

$$D_{applied}^{\text{tank}} = C_{pest}^{\text{tank}} \times \text{Application}_{\text{rate}} \times 1\text{e}^{-9} \quad \text{(S6)}$$

where C_{pest}^{tank} is pesticide concentration measured in the tank (ng L^{-1}) and Application_rate is the volumetric application rate (95 L ha^{-1}) . The factor 1e^{-9} is used to obtain g ha^{-1} from ng ha^{-1}. Finally, the target dose was obtained from farmer's information with the equation:

$$D_{applied}^{\text{farmer}} = V_{product} \times C_{product} \quad \text{(S7)}$$

where $V_{product}$ is the volume of commercial product applied (1.3 L ha^{-1}) and $C_{product}$ is the active ingredient concentration in commercial product solution (375 g L^{-1}). The three methods provided application dose that agreed remarkably well (**Supplementary Table 1**). A Student t-test showed no rejection of the null hypothesis (t = 0.11, df = 5.4, p-value = 0.91), meaning that the means were not significantly different between dose application estimates using filters, tank and farmer estimates.

Supplementary Table 1. Pesticide application dose statistics.

Method	Dose	std. dev	Analysis replicates
-	g ha^{-1}	g ha^{-1}	-
Farmer target dose	487		
Dose estimated from tank sampling	544	59	4
Dose estimated from filter papers placed above the canopy	540	54	9
Ground dose estimated from filter papers placed at the ground	374	36	9

Supplementary section C: GC-MS chromatograms analysis details

Regarding possible interferences, the mass spectrometer used in this study allows to perform simultaneously an acquisition in SCAN mode and an acquisition in SIM mode on the same sample, thanks to the fast electronics speed of the spectrometer. After a software processing of the acquired signal, several chromatograms can be obtained according to the objectives of analysis (quantification or identification): (1) the TIC SCAN, or *Total Ion Current chromatogram*, which was an acquisition on a pre-defined mass range from 30 to 380 m/z ; (2) the TIC SIM or Total Ion chromatogram Selected Ion Monitoring, which was an acquisition on specific ion 266 m/z in the case of this study.

As shown in a typical example chromatogram in we observed no interference in filter extraction solution in the TIC SCAN (**Supp. Figure C1-a**). On the contrary many compounds were observed around the Chlorothalonil peak in thermo-desorbed air samples in the TIC SCAN (**Supp. Figure C1-b**). That is why, to ensure the robustness of our results and reduce possible interferences, we used the TIC SIM to quantify the Chlorothalonil peak, which allowed for a good selectivity during the whole campaign.

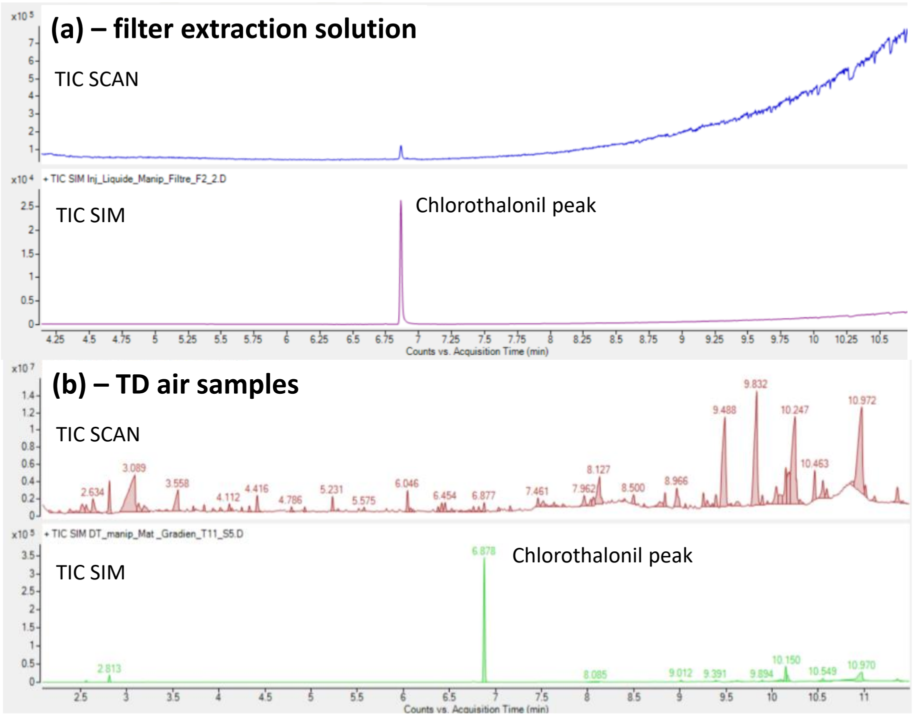

Supplementary Figure C1. Example of chromatograms used to quantify Chlorothalonil in (a) the filter extraction solution, and (b) thermo-desorbed cartridges air samples. Two chromatograms were used: the TIC SCAN "Total Ion Current chromatogram: acquisition on a pre-defined mass range from 30 to 380 m/z SCAN, was used to check if there was any interference. The TIC SIM "Total Ion chromatogram Selected Ion Monitoring : acquisition on a chlorothalonil specific ion 266 m/z, was used to quantify the Chlorothalonil peak area.

Supplementary Figures

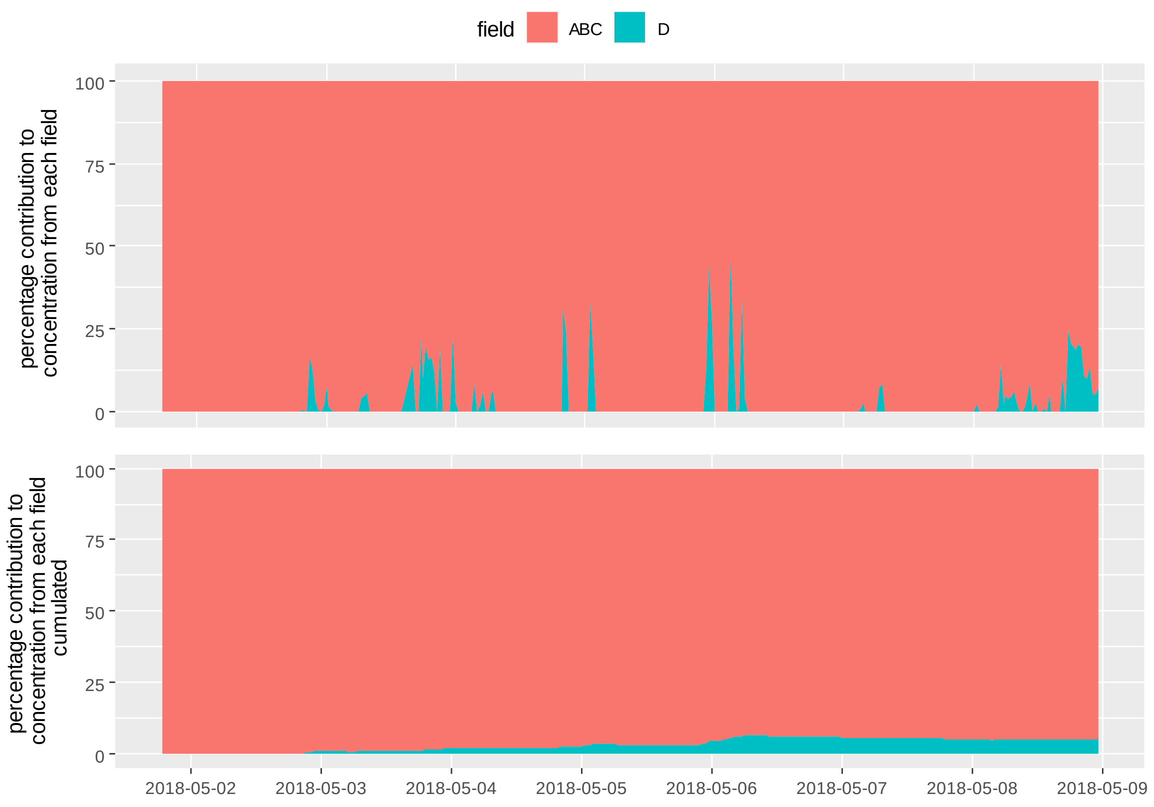

Supplementary Figure 1. Percentage contribution from fields D and ABC to the concentration footprint at the PTR-QI-TOF-MS measurement location over the campaign's last week after the field was applied. Assuming a similar source from each field, the cumulated contribution from field D to the chlorothalonil concentration at the PTR-QI-TOF-MS location would represent less than 5.5% (bottom graph). The contribution from field D is scarce in time and primarily high during nights (top graph). When multiplied by a modelled flux simulated over that period and starting from the day of application of field D, we estimate that field D's contribution to the measured concentration by the PTR-QI-TOF-MS would be less than 2%.

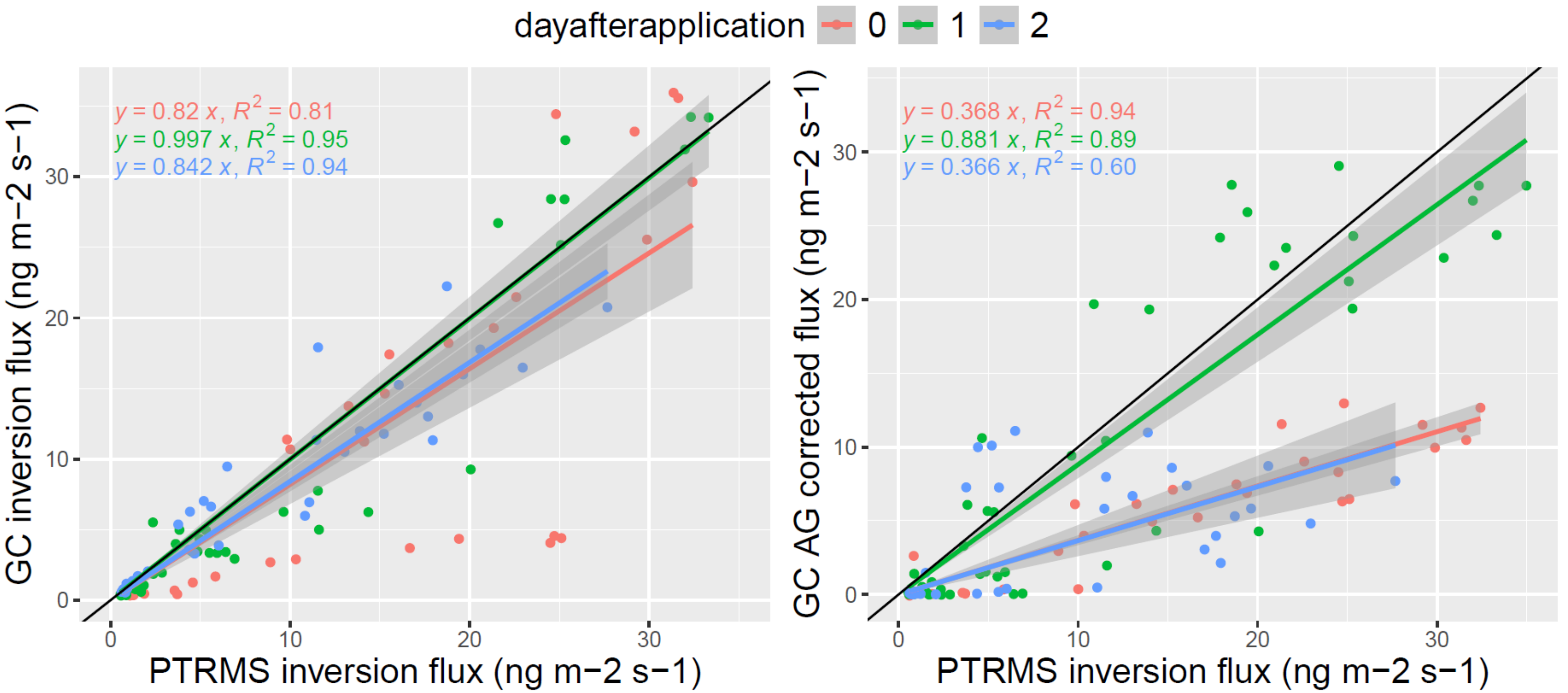

Supplementary Figure 2a. GC inversion (left) and GC corrected gradient (right) chlorothalonil fluxes versus PTR-QI-TOF-MS inversion flux. The GC inversion flux was computed using the inverse dispersion method, and the chlorothalonil concentration was measured by the GC-MS at the GC-MS location at 1.25 m height above ground. The aerodynamic method was corrected to account for the application exclusion zone by using a footprint model (see methods). The linear fits are shown for each day after application.

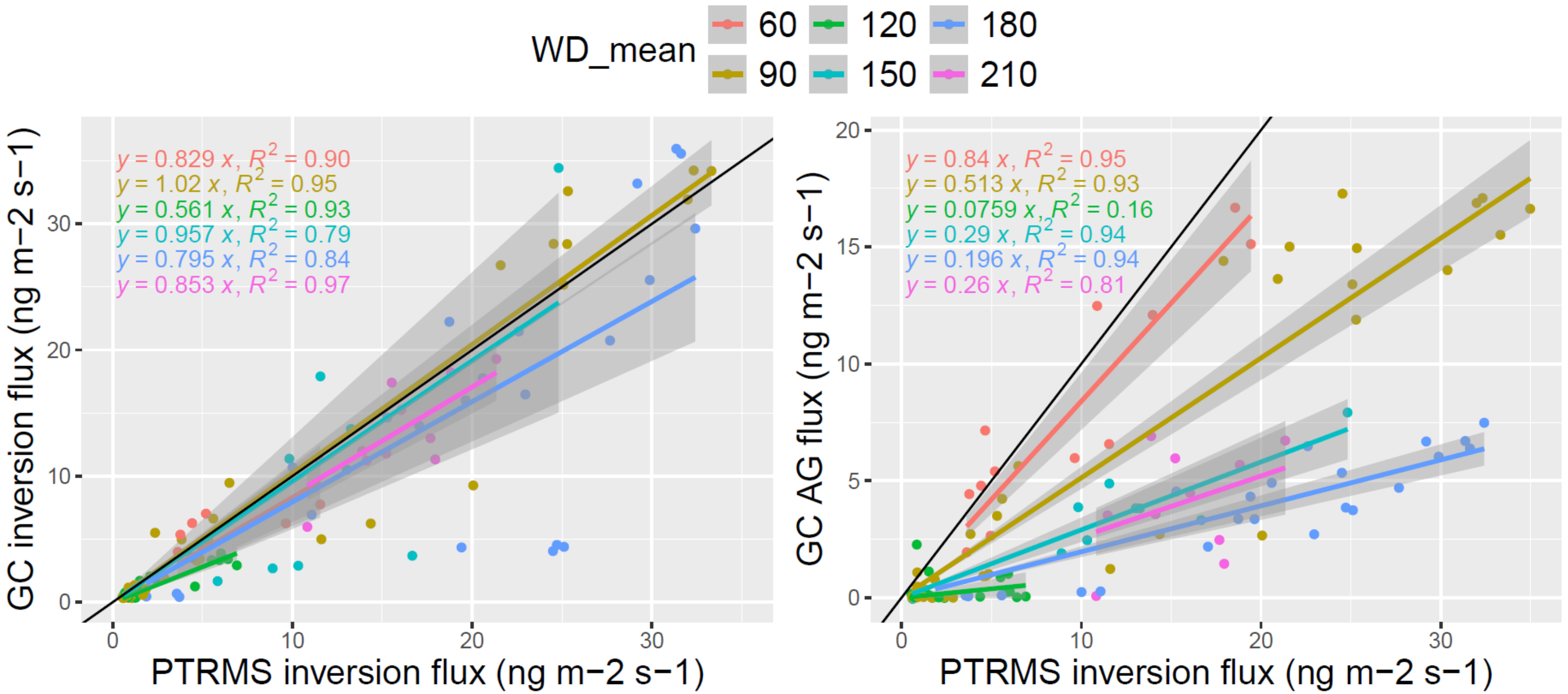

Supplementary Figure 2b. Same as Supp. Fig 2a, but grouped by 30 degrees wind sectors. WD_mean is the start of the wind sector. The effect of the exclusion zone on the aerodynamic gradient flux is clear: when the wind was blowing from that zone, the GC AG flux was much smaller than the flux obtained by PTRMS inversion. There is no effect of the wind sectors on the flux obtained by inversion of the GC measurements at the GC mast. This difference in sensitivity to the wind direction between the GC inversion and GC aerodynamic gradient flux is explained by the difference between the flux footprint and the concentration footprint. Indeed, the inversion method is sensitive to the concentration footprint which is much larger than the flux footprint over which the aerodynamic gradient is sensitive.

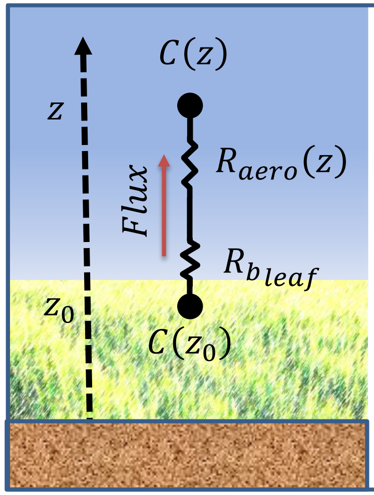

$$Flux = \frac{C(z_0) - C(z)}{R_{aero}(z) + R_{b_{leaf}}}$$

$$C(z_0) = C(z_{ref}) + F \times \left(R_b + R_a(z_{ref})\right)$$

$$p_{vap} = C \times 10^{-9} \times \frac{R\,(T_{air} + 273.15)}{M_{Chl}}$$

Supplementary Figure 3. The resistance scheme concept and equations and how this is used to compute the vapour pressure at z_0.

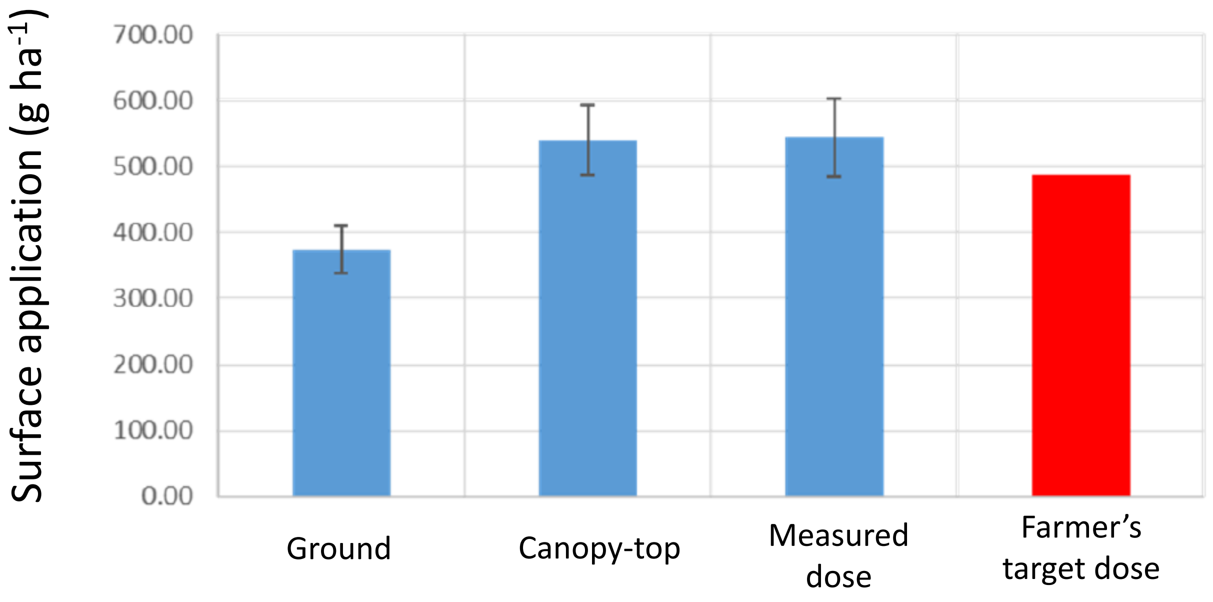

Supplementary Figure 4. Chlorothalonil dose applied as estimated by different means. Ground and canopy-top were estimated using filters (n = 12), while the measured dose was estimated by measuring the tank concentration (n = 4). The farmer’s target dose is the dose targeted by the farmer. See Supp section B for details.

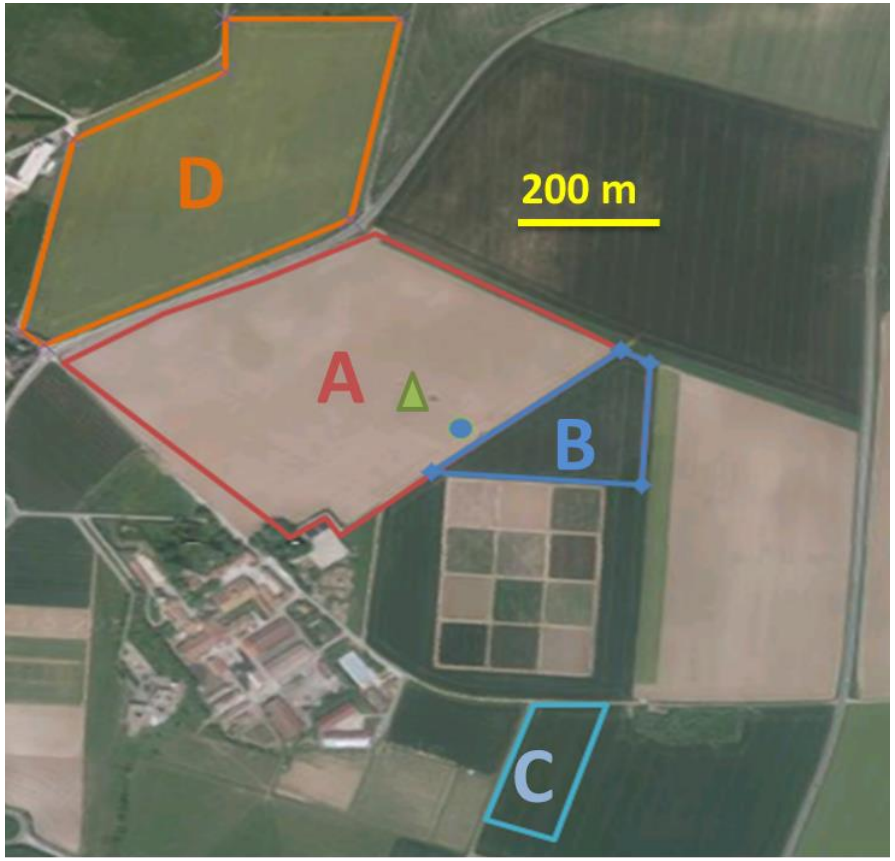

Supplementary Figure 5. Experimental site (A). Applied fields are shown in colours with letters A-D. The circle shows the PTR-QI-TOF-MS sampling location, and the triangle shows the TD-GC-MS gradient sampling location. A, B and C fields were applied on the same day (16/04/2018), while field D was applied on 29/04/2018. The map behind the figure was taken from Google Maps^{TM} the 01/04/2022.

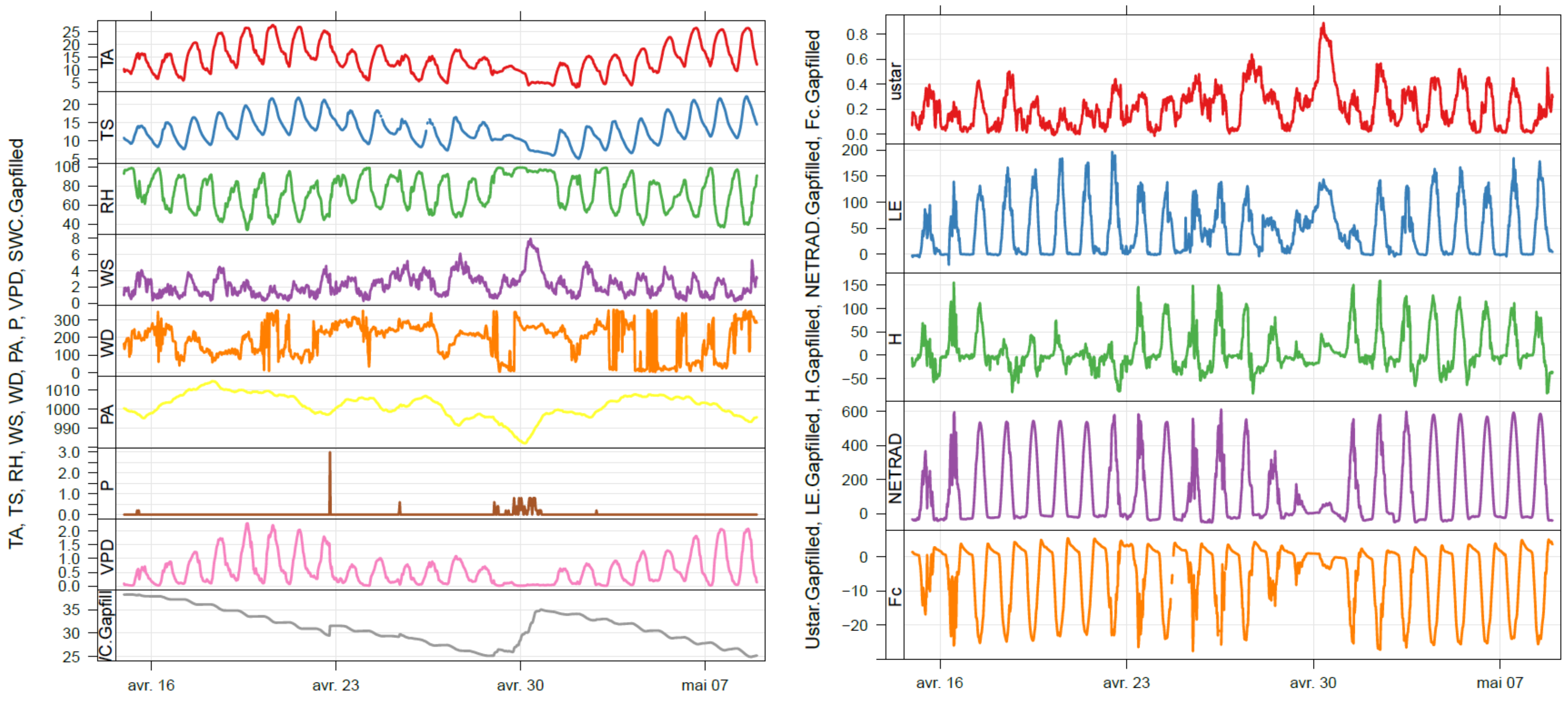

Supplementary Figure 6. Meteorological conditions during the experiment. Air temperature (TA, °C), soil surface temperature (TS, °C), relative humidity '(RH, %), wind speed (WS, m s-1), wind direction (WD, deg/N), air pressure (PA, mbar), precipitation (P, mm), vapour pressure deficit (VPD, kPa), volumetric soil water content at 10 cm depth (SWC, %), friction velocity (ustar, ms s-1), latent heat flux (LE, W m-2), sensible heat flux (H, W m-2), net radiation (NETRAD, W m-2), CO2 flux (Fc, µmon m-2 s-1).

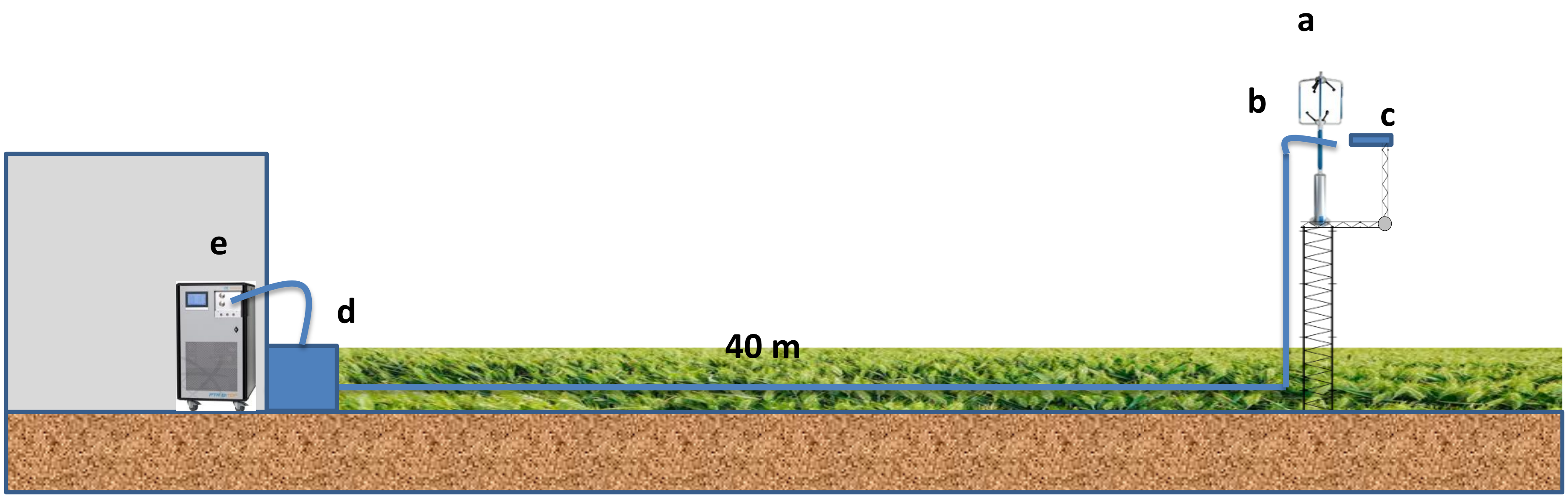

Supplementary Figure 7. Field PTR-QI-TOF-MS set up. a - ultrasonic anemometer. b – PTR-QI-TOF-MS sampling line. c – thermodesorption cartridge for GC-MS analysis. d – Pump for sampling line. e – PTR-QI-TOF-MS in its temperature-controlled field lab.

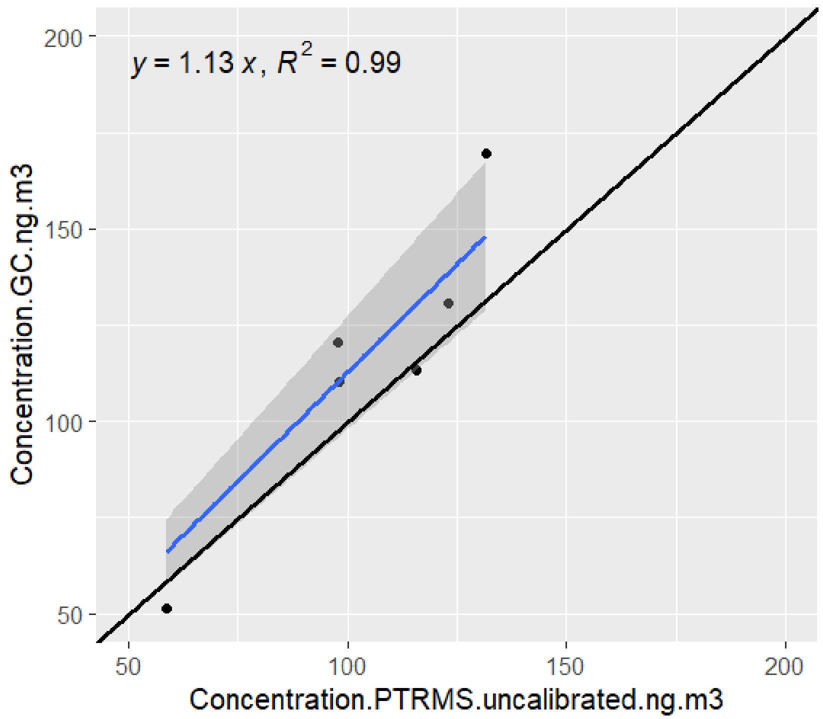

Supplementary Figure 8. PTR-Qi-TOF-MS calibration curve: calibrated TD-GC–MS versus uncalibrated PTR-Qi-TOF-MS chlorothalonil concentrations measured in the field during the first three days of the experimental campaign. The equation shown was used to calibrate the PTR-Qi-TOF-MS. The p-value of the linear regression, which is the probability that the slope is zero (the null hypothesis), computed using the *lm*() function in R forcing the intercept to 0 was p.value = 6e-6. The standard error on the slope was 0.057. The shaded area shows the 95% confidence interval. Each point corresponds to a 3h period of the TD-GC-MS sampling interval. The PTR-Qi-TOF-MS 30 min data were averaged over this interval. If any 30-min PTRMS data was missing within the 3h interval the average was discarded.

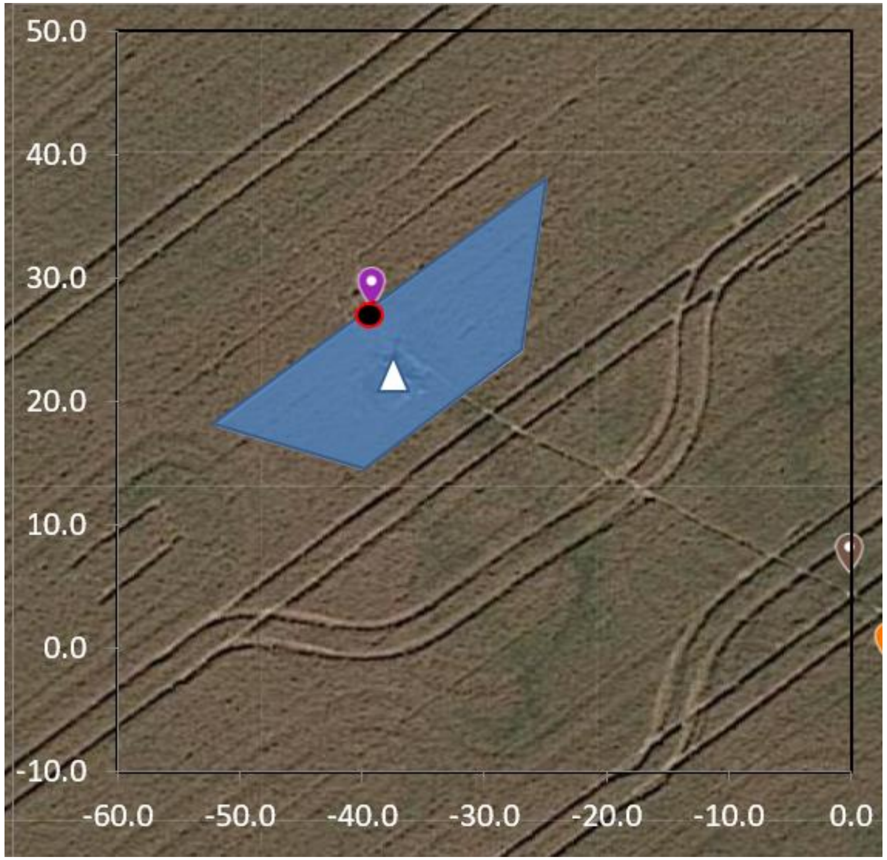

Supplementary Figure 9. Application exclusion zone (blue surface) and location of the GC aerodynamic gradient mast (purple locator and black and red circle). The tractor paths can be seen on the Google map photograph. The change in the path to avoid the 5 m tall Eddy Covariance mast (white triangle) can be seen on the satellite image. This path resulted in a application exclusion zone (in blue) where no pesticide have been applied. This area width was evaluated as half the application swath (24 m), resulting in an approximately 200 m^2 area located at the south-easterly side of the GC mast. Scales on the image are given in meters. The map behind is taken from Google Earth™ archive and was taken the 26 June 2018.

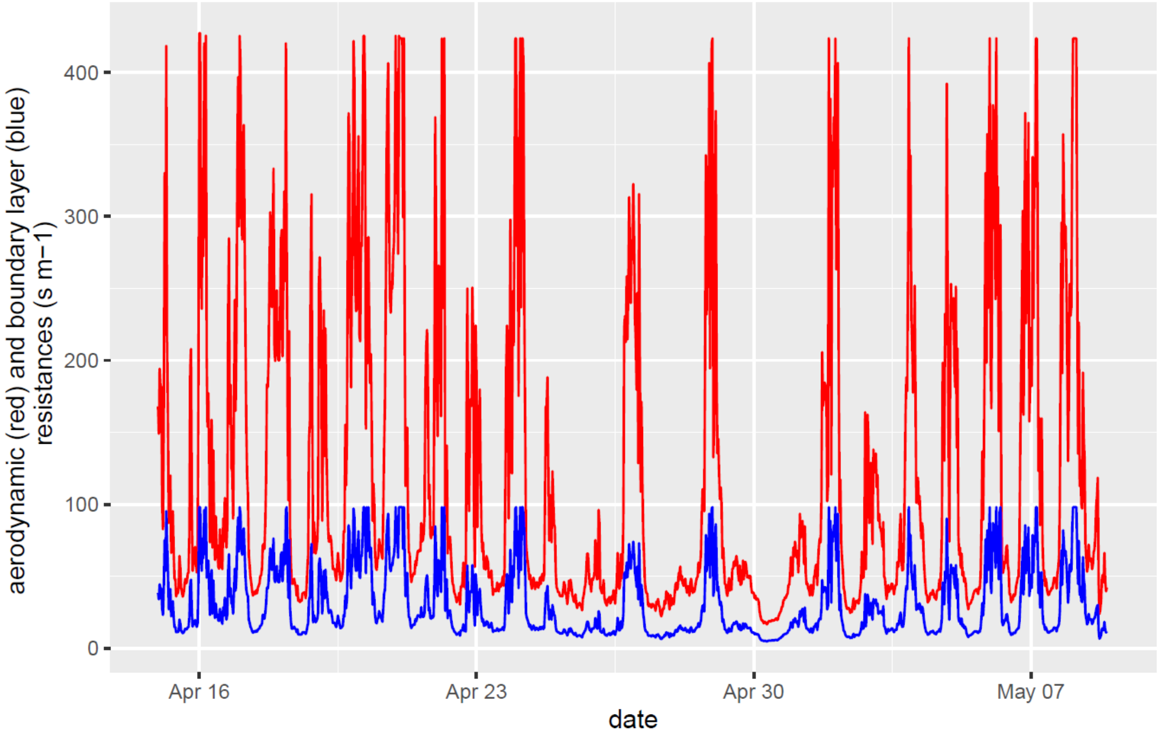

Supplementary Figure 10. Aerodynamic $R_a(z_{ref})$ (in blue), and canopy boundary layer R_b (in red) resistances that are used to compute the surface concentrations and vapour pressures as a function of time.

Supplementary tables

Supplementary Table 1. Chlorothalonil usage in France from 2009 to 2016.

year	use (T per year)
2009	984
2010	1213
2011	1320
2012	1084
2013	1091
2014	1293
2015	1448
2016	1710

Source: https://www.anses.fr/fr/system/files/Fiche_PPV_Chlorothalonil.pdf

Supplementary material - references

Kaimal, J. C. and Finnigan, J. J.: Atmospheric Boundary Layer Flows, Their structure and measurement., Oxford University Press., New York, 289 pp.1994.

Loubet, B., Milford, C., Sutton, M. A., and Cellier, P.: Investigation of the interaction between sources and sinks of atmospheric ammonia in an upland landscape using a simplified dispersion-exchange model, Journal of Geophysical Research-Atmospheres, 106, 24183-24195, Doi 10.1029/2001jd900238, 2001.

Loubet, B., Carozzi, M., Voylokov, P., Cohan, J.-P., Trochard, R., and Génermont, S.: Evaluation of a new inference method for estimating ammonia volatilisation from multiple agronomic plots, Biogeosciences, 15, 3439-3460, 10.5194/bg-15-3439-2018, 2018.

Philip, J. R.: The Theory of Local Advection .1., Journal of Meteorology, 16, 535-547, Doi 10.1175/1520-0469(1959)016<0535:Ttolai>2.0.Co;2, 1959.

Sutton, O. G.: A Theory of Eddy Diffusion in the Atmosphere, Proceedings of the Royal Society of London. Series A, 135, 143-165, 10.1098/rspa.1932.0025, 1932.
